# Impact of Simulated Risk Factor Management on Cardiovascular Disease and Costs in Type 2 Diabetes Using the ABC Model

**DOI:** 10.1111/dom.70755

**Published:** 2026-04-13

**Authors:** Lee‐Ling Lim, Eric S. H. Lau, Juliana N. M. Lui, Linong Ji, A. G. Unnikrishnan, Sirinart Sirinvaravong, Siew‐Pheng Chan, Soo Lim, Andrea O. Y. Luk, Juliana C. N. Chan

**Affiliations:** ^1^ Department of Medicine, Faculty of Medicine University Malaya Kuala Lumpur Malaysia; ^2^ Department of Medicine and Therapeutics The Chinese University of Hong Kong, Prince of Wales Hospital Shatin Hong Kong SAR; ^3^ Asia Diabetes Foundation Hong Kong SAR; ^4^ Baker Heart and Diabetes Institute Melbourne Australia; ^5^ Hong Kong Institute of Diabetes and Obesity The Chinese University of Hong Kong, Prince of Wales Hospital Shatin Hong Kong SAR; ^6^ Department of Endocrinology and Metabolism Peking University People's Hospital Peking China; ^7^ Department of Endocrinology, Chellaram Diabetes Institute Pune Maharashtra India; ^8^ Department of Medicine, Faculty of Medicine Siriraj Hospital Mahidol University Bangkok Thailand; ^9^ Department of Internal Medicine Seoul National University Bundang Hospital, Seoul National University College of Medicine Seongnam South Korea

**Keywords:** cardiovascular disease, glycaemic control, health economics, real‐world evidence, risk prediction, type 2 diabetes

## Abstract

**Aims:**

We evaluated the impact of scenario‐based multifactorial control on the 3‐year incidence of fatal/non‐fatal cardiovascular disease (CVD) and associated healthcare costs in Asian patients with type 2 diabetes (T2D).

**Materials and Methods:**

We applied the validated ‘ABC’ model to simulate the 3‐year impact of attaining multiple treatment targets (HbA_1c_ < 7%, blood pressure [BP] < 130/80 mmHg, risk‐stratified LDL‐C target [ABC], smoking cessation and weight reduction) on the incidence of fatal/non‐fatal CVD (ischaemic heart disease and stroke) in patients with T2D enrolled from 11 Asian countries/jurisdictions in the Joint Asia Diabetes Evaluation (JADE) Register. We estimated the costs of these simulated CVD events and potential cost savings with multifactorial intervention.

**Results:**

Amongst 103 958 patients with T2D (53.4% men, 12.0% current smokers, mean [SD] age 57.6 [11.8], HbA_1c_ 7.96 [1.87], systolic BP 132.0 [17.4] mmHg, LDL‐C 2.6 [0.95] mmol/L, BMI 26.2 [4.5] kg/m^2^), 17 151 (16.5%) had prior CVD and 86 807 (83.5%) had no CVD at baseline. In the CVD group, 1.5% achieved all three ABC targets and 5.1% in the nonCVD group at baseline. In the CVD group, the estimated incidence rate for CVD was 61.60 per 1000‐person‐years (PY), and 11.65 per 1000‐PY in the non‐CVD group. Simulated achievement of all three ABC targets reduced the incidence rates by 35.1%–40.01% per 1000‐PY in the CVD group and by 30.2%–8.13% per 1000‐PY in the non‐CVD group. In China, these scenario‐based simulations might avert 1 177 682 CVD events per year with a potential saving of US$ 11 292 823 476.

**Conclusions:**

Multifactorial management of cardiometabolic risk factors may have a substantial impact on CVD prevention, with cost savings in patients with T2D.

## 1 Introduction

Cardiovascular disease (CVD), including ischaemic heart disease (IHD) and stroke, is the leading cause of death amongst individuals with type 2 diabetes (T2D) [1]. Effective control of cardiometabolic risk factors is critical for primary and secondary prevention of CVD [1, 2]. Key modifiable risk factors include elevated glycated haemoglobin (HbA_1c_), blood pressure (BP), low‐density lipoprotein cholesterol (LDL‐C) and adiposity [1, 3, 4, 5, 6, 7, 8]. In the STENO‐2 trial, intensified, target‐driven multifactorial intervention reduced CVD and microvascular events by 50% compared with conventional care [9]. In meta‐analyses, lowering HbA_1c_ by 0.9% reduced CVD risk by 9% [10], lowering LDL‐C reduced CVD risk proportionally with 1 mmol/L associated with 21% risk reduction [11], and lowering systolic BP by 10 mmHg reduced CVD risk by 11% [12]. In a nationwide observational study, achievement of multiple cardiometabolic risk factor targets in T2D was associated with attenuated risk of CVD and death similar to that in the general population [13]. Control of cardiometabolic risk factors in T2D was also associated with improved health‐related quality of life (HRQoL) [14]. Supported by this large body of evidence, integrated, multifactorial management is a key component of practice guidelines aimed at reducing complications and improving HRQoL in individuals with T2D [9, 10, 12, 15, 16, 17, 18, 19, 20].

There are substantial care gaps in real‐world practice, particularly in low‐ and middle‐income countries (LMICs), where attainment of guideline‐recommended targets remains suboptimal [21, 22, 23]. The economic burden of T2D is enormous with reduced workforce productivity and increased healthcare costs, largely driven by hospitalizations due to CVD and other complications [21, 22, 23, 24, 25, 26]. Most adults with T2D live in LMICs [27], where rapid lifestyle and dietary changes, physical inactivity, low health literacy, social disparity and limited access to care contribute to the rising prevalence of diabetes and its complications [2, 3]. In retrospective claims‐based analyses, improved glycemic control and prevention of CVD‐related hospitalizations were associated with significant cost savings [25, 28, 29, 30, 31], calling for urgent preventive strategies to preserve health and economic well‐being [25].

Quantification of the clinical and economic impact of improving risk factor control will inform practice and policies. Based on the prospective data from the Hong Kong Diabetes Register (HKDR) in Chinese patients with T2D, we developed the mathematical ‘ABC’ model which used routine measures including HbA_1c_, BP and LDL‐C to predict 3‐year CVD incidence. The model was externally validated using published data from the Swedish National Diabetes Register (NDR) [5]. Using published prevalence data, we simulated the impact of implementing integrated care to control multiple risks which averted a large number of CVD events and healthcare costs in mainland China [5].

In this study, we used a scenario‐based ABC modelling framework to provide region‐specific estimates for CVD incidence rates, number of CVD events and associated costs in Asian patients with T2D enrolled in the Joint Asia Diabetes Evaluation (JADE) Register. We estimated the 3‐year impact of multifactorial risk factor control on the incidence of fatal and non‐fatal CVD events, including IHD and stroke, as well as related healthcare costs, stratified by the presence of CVD at baseline. Scenario‐based simulation included attainment of targets of three ABC risk factors (HbA_1c_ < 7%, BP < 130/80 mmHg, risk‐stratified LDL‐C), smoking cessation and body weight reduction.

## 2 Methods

### 2.1 Study Design and Population

This simulation‐modelling study utilised the JADE Register established through the web‐based JADE technology [32]. The JADE platform includes protocols to enable systematic data collection during assessments at baseline and every 12–14 months [20, 33, 34]. From November 2007 to December 2019, 116 000 patients with diabetes (both type 1 diabetes [T1D] and T2D) were enrolled by over 300 physicians across Asia [14, 33, 35]. Guided by a case report form, sociodemographic data, disease duration, lifestyle/self‐management, comorbidities, current medications, hypoglycemia and diabetes‐related complications were documented with collection of fasting blood and random urinary samples for measurement of risk factors [14, 36]. Data were deidentified upon entry, and all patients provided written informed consent. The study was approved by CUHK‐NTEC Clinical Research Ethics Committee, Hong Kong SAR.

Patients with T1D, defined by ketotic presentation or continuous insulin requirement within 12 months of diagnosis, were excluded. Diagnosis of T2D was based on criteria of the American Diabetes Association [20].

After excluding patients with missing data, 103 958 patients with T2D aged ≥ 20 years from 11 Asian countries/jurisdictions (Mainland China, Hong Kong SAR, India, Indonesia, South Korea, Malaysia, the Philippines, Singapore, Taiwan, Thailand and Vietnam) were included in the analysis.

### 2.2 Statistical Analysis

#### 2.2.1 Development of the ABC Model for Patients With Diagnosed T2D

The ABC mathematical model estimates the incidence of CVD, including IHD and stroke, for up to 3 years in patients aged 20–79 years with T2D [5]. Peripheral vascular disease was not assessed due to few events in the training dataset. The ABC model was trained with the HKDR (1994–2007) and the JADE‐Hong Kong (JADE‐HK 2007–2015) datasets with major events based on International Classification of Diseases (ICD) codes (censored in 2017). Validation was performed with event rates from the Hong Kong Diabetes Database (HKDD) [37], and externally validated using the Swedish NDR [13]. The rate ratios between the predicted and observed rates for IHD and stroke events were close to 1, supporting the validity of the model in Asian and European patients with T2D [5]. Detailed methodology is available in the Lancet Commission Report on Diabetes [5].

#### 2.2.2 Applying the ABC Model to the JADE Register Dataset

Modelled treatment targets were defined as: HbA_1c_ < 7%, systolic BP (SBP)/diastolic BP (DBP) < 130/80 mmHg and LDL‐C < 2.6 mmol/L (low to moderate CV risk), < 1.8 mmol/L (high CV risk) and < 1.4 mmol/L (very high CV risk) [15, 38], using the European Society of Cardiology CV risk stratification [15]. Asian criteria were used to define overweight (BMI: 23.0–24.9 kg/m^2^) and obesity (≥ 25 kg/m^2^) [39]. We excluded patients with missing data for any one of these parameters needed to estimate event rates. We did not perform any data imputation or model calibration. We applied the original ABC risk equation to the JADE cohort and simulated the incidence of CVD based on baseline risk factors and the assumption of treatment to ABC targets. Meta‐analyses [10, 11, 12] had confirmed that HbA_1c_, BP and LDL‐C control each independently reduce CVD risk, regardless of other risk factors. Therefore, we simulated the effects of achieving different targets on 3‐year CVD incidence and the number of CVD events averted compared to baseline. The simulated incidence rates were expressed by 1000‐person‐years (PY). Additionally, we estimated the effects of modelled reduction of 3%–15% body weight in patients classified as overweight or obese. We modelled smoking cessation in active smokers at baseline.

Risk reduction was expressed with 95% confidence intervals estimated using the bootstrapping method. The simulated reductions in the 3‐year incidence of CVD, IHD, or stroke were estimated by reducing HbA_1c_, BP, LDL‐C, BMI and smoking cessation.

### 2.3 Cost Analysis

We obtained the number of individuals with diabetes per country from the 2021 International Diabetes Federation (IDF) Atlas [27]. We simulated the 3‐year incidence of CVD by applying the varying distribution of risk factors from five countries/jurisdictions with at least 5000 patients in the JADE Register. We estimated the potential number of CVD events averted by modelled reduction of ABC risk factors to target, body weight reduction and smoking cessation.

Hong Kong has a highly subsidised healthcare system, and the majority of patients with chronic diseases such as diabetes received outpatient and inpatient care in publicly funded healthcare facilities. The costs associated with healthcare utilisation are available from the Government Gazette, with unit costs specific to the year of analysis, making adjustments for inflation unnecessary. These territory‐wide healthcare utilisation data, together with publicly available healthcare costs, enabled the estimation of the costs of major outcomes in T2D based on ICD codes [40, 41]. In this analysis, we used the World Bank Purchasing Power Parity (PPP) ratio and PPP exchange rate formula to estimate the cost of CVD events for each country/jurisdiction using Hong Kong as reference [40, 41]. The World Bank PPP adjusts for differences in price levels and cost of living, reflecting the purchasing power of money rather than its nominal value on currency markets. The estimate was based on real income, poverty and economic sizes, and is a preferred index for cost comparisons [42, 43]. We did not adjust for complications other than CVD at baseline and assumed the cost to be a combination of public and private direct medical costs. Equations used in the cost analysis are as follows:

-
$$\begin{array}{c} \text{Cost of complication}=\left(\frac{\mathrm{PPP}\;\text{of country}}{\mathrm{PPP}\;\text{Hong Kong}\;\left[\mathrm{HK}\right]}\right)\\ \;\times \text{Cost of complication in}\;\mathrm{HK} \end{array}$$
-
Number of events=Number with diabetes×Incidence
-
$$\begin{array}{c} \text{Cost difference}=\left(\text{Cost of complication}\right)\\ \;\times \left(\text{Base case}-\text{Number of events}\right) \end{array}$$

## 3 Results

### 3.1 Validation of the ABC Model

The HKDR and HKDD cohorts had longer disease duration, lower BMI and fewer smokers than the Swedish NDR database [5]. Predicted and observed event rates were similar for IHD and stroke amongst the HKDR, HKDD and Swedish NDR cohorts with ratios of observed to predicted events of 0.999–1.253. These near‐1 ratios supported the validity of the ABC model in both Asian and European populations [5]. Table S1 shows prevalence of diabetes per country/jurisdiction (IDF 2021). Table S2 shows the cost of CVD, PPP conversion factor and exchange rate per country/jurisdiction.

### 3.2 Baseline Clinical Profiles in the JADE Register

The CVD group had older age (63.5 vs. 56.4 years), male preponderance (60.9% vs. 51.9%), longer disease duration (10 vs. 5 years), higher SBP (136 vs. 131 mmHg) and lower mean LDL‐C (2.38 vs. 2.65 mmol/L) than the non‐CVD group. Both groups had similar usage of glucose lowering drugs, with the CVD group being more likely to be on organ‐protective drugs and less likely to attain BP target (24.3% vs. 28.9%) than the non‐CVD group. Amongst statin‐treated patients, 12.3% in the CVD group and 31.5% in the non‐CVD group met LDL‐C targets. In both groups, more than one in three patients had at least one ABC risk factor not on target, with 5.1% in the non‐CVD group and 1.5% in the CVD group meeting all three ABC targets and 35.3% in the non‐CVD group and 46.3% in the CVD group not meeting any ABC target (Table 1).

**TABLE 1:** Clinical profiles of patients with T2D in the JADE Register (2007–2019), stratified by CVD status at baseline.

	Number of patients with data	All patients	Number of patients with data	Patients with no prior CVD	Number of patients with data	Patients with prior CVD
Total number of persons, *n*	—	103 958	—	86 807	—	17 151
Mean age, years (SD)	103 958	57.6 (11.8)	86 807	56.4 (11.6)	17 151	63.5 (10.8)
Male sex, *n* (%)	103 951	55 462 (53.4)	86 800	45 018 (51.9)	17 151	10 444 (60.9)
Median disease duration, years (IQR)	102 083	6 (2–12)	85 077	5 (2–11)	17 006	10 (4–16)
Non‐smoker, *n* (%)	102 111	74 002 (72.5)	85 144	63 272 (74.3)	16 967	10 730 (63.2)
Ex‐smoker, *n* (%)	102 111	15 852 (15.5)	85 144	11 563 (13.6)	16 967	4289 (25.3)
Current smoker, *n* (%)	102 111	12 257 (12.0)	85 144	10 309 (12.1)	16 967	1948 (11.5)
Prior IHD, *n* (%)	103 958	9957 (9.6)	86 807	0 (0.0)	17 151	9957 (58.1)
Prior stroke, *n* (%)	103 958	3958 (3.8)	86 807	0 (0.0)	17 151	3958 (23.1)
Mean BW, kg (SD) (male)	54 175	73.1 (13.4)	43 852	73.2 (13.6)	10 323	72.6 (12.3)
Mean BW, kg (SD) (female)	46 820	63.2 (13.1)	40 292	63.3 (13.3)	6528	62.8 (12.1)
Mean Waist, cm (SD) (male)	46 865	92.3 (11.2)	37 317	92.1 (11.2)	9548	92.9 (11.4)
Mean Waist, cm (SD) (female)	38 423	88.4 (12.1)	32 640	88.3 (12.1)	5783	89.1 (12.5)
Mean BMI, kg/m^2^ (SD)	98 158	26.2 (4.54)	81 472	26.2 (4.58)	16 686	26.3 (4.33)
Mean HbA_1c_, (SD)	90 090	7.96 (1.87)	74 601	7.97 (1.89)	15 489	7.92 (1.76)
Mean SBP (mmHg), (SD)	101 606	132 (17.4)	84 682	131 (17)	16 924	136 (18.4)
Mean DBP (mmHg), (SD)	101 428	78.7 (9.69)	84 524	78.8 (9.6)	16 904	78.4 (10.1)
Mean LDL‐C, mmol/L (SD)	83 267	2.6 (0.954)	68 754	2.65 (0.958)	14 513	2.38 (0.899)
Mean HDL‐C, mmol/L (SD)	83 872	1.19 (0.386)	69 249	1.2 (0.391)	14 623	1.17 (0.364)
Median TG, mmol/L (IQR)	88 702	1.55 (1.10–2.15)	73 289	1.55 (1.10–2.17)	15 413	1.54 (1.10–2.08)
HbA_1c_ < 7%, *n* (%)	90 090	31 226 (34.7)	74 601	26 070 (34.9)	15 489	5156 (33.3)
BP < 130/80 mmHg, *n* (%)	101 457	28 567 (28.2)	84 547	24 458 (28.9)	16 910	4109 (24.3)
LDL‐C < CV‐risk based target, *n* (%)	71 431	17 421 (24.4)	56 918	15 927 (28.0)	14 513	1494 (10.3)
LDL‐C < 2.6 mmol/L (low‐moderate CV‐risk), *n* (%)	24 714	12 587 (50.9)	24 714	12 587 (50.9)	0	0 (0.0)
LDL‐C < 1.8 mmol/L (high CV‐risk), *n* (%)	17 192	2154 (12.5)	17 192	2154 (12.5)	0	0 (0.0)
LDL‐C < 1.4 mmol/L (very high CV‐risk), *n* (%)	29 525	2680 (9.1)	15 012	1186 (7.9)	14 513	1494 (10.3)
On 0 target, *n* (%)	70 339	26 334 (37.4)	56 475	19 915 (35.3)	13 864	6419 (46.3)
On 1 target, *n* (%)	70 339	27 544 (39.2)	56 475	22 152 (39.2)	13 864	5392 (38.9)
On 2 targets, *n* (%)	70 339	13 367 (19.0)	56 475	11 518 (20.4)	13 864	1849 (13.3)
On all 3 targets, *n* (%)	70 339	3094 (4.4)	56 475	2890 (5.1)	13 864	204 (1.5)
On OGLD only, *n* (%)	103 958	70 781 (68.1)	86 807	60 307 (69.5)	17 151	10 474 (61.1)
On HbA_1c_ target whilst on OGLD only, *n* (%)	61 047	24 111 (39.5)	51 621	20 376 (39.5)	9426	3735 (39.6)
On insulin ± OGLD, *n* (%)	103 958	20 979 (20.2)	86 807	16 145 (18.6)	17 151	4834 (28.2)
On HbA_1c_ target whilst on insulin, *n* (%)	19 036	3141 (16.5)	14 631	2402 (16.4)	4405	739 (16.8)
On statin, *n* (%)	88 398	39 224 (44.4)	74 300	29 480 (39.7)	14 098	9744 (69.1)
On LDL‐C target whilst on statin, *n* (%)	29 983	7786 (26.0)	21 336	6719 (31.5)	8647	1067 (12.3)
On RASi, *n* (%)	89 400	36 521 (40.9)	75 053	27 771 (37.0)	14 347	8750 (61.0)
On BP target whilst on RASi, *n* (%)	36 030	8323 (23.1)	27 362	6306 (23.0)	8668	2017 (23.3)
On AGI, *n* (%)	90 383	5590 (6.2)	74 797	4591 (6.1)	15 586	999 (6.4)
On DPP4i, *n* (%)	90 383	17 377 (19.2)	74 797	14 481 (19.4)	15 586	2896 (18.6)
On glinides, *n* (%)	90 383	1149 (1.3)	74 797	928 (1.2)	15 586	221 (1.4)
On metformin, *n* (%)	90 383	60 421 (66.8)	74 797	50 728 (67.8)	15 586	9693 (62.2)
On SGLT2i, *n* (%)	90 383	408 (0.5)	74 797	306 (0.4)	15 586	102 (0.7)
On sulphonylurea, *n* (%)	90 383	40 637 (45.0)	74 797	33 856 (45.3)	15 586	6781 (43.5)
On thiazolidinedione, *n* (%)	90 383	6135 (6.8)	74 797	5176 (6.9)	15 586	959 (6.2)
On GLP1‐RA, *n* (%)	90 384	297 (0.3)	74 798	245 (0.3)	15 586	52 (0.3)

*Note*: LDL‐C < 2.6 mmol/L (if low/moderate CV risk) or LDL‐C < 1.8 mmol/L (if high CV risk) or LDL‐C < 1.4 mmol/L (if very high CV risk). Abbreviations: AGI, alpha‐glucosidase inhibitor; BMI, body‐mass index; BP, blood pressure; BW, body weight; CV, cardiovascular; CVD, cardiovascular disease; DBP, diastolic blood pressure; DPP4i, dipeptidyl peptidase‐4 inhibitor; GLP1‐RA, glucagon‐like peptide‐1 receptor agonist; HbA_1c_, glycated haemoglobin A1c; HDL‐C; high‐density lipoprotein‐cholesterol; IHD, ischemic heart disease; IQR, interquartile range; LDL‐C, low‐density lipoprotein‐cholesterol; OGLD, oral glucose lowering drug; RASi, renin angiotensin system inhibitors; SBP, systolic blood pressure; SD, standard deviation; SGLT2i, sodium‐glucose co‐transporter 2 inhibitor; TG, triglyceride.

Only for patients with complete relevant data, 3‐year CVD incidence was predicted. Baseline characteristics of patients with and without prediction of 3‐year CVD incidence are provided in Table S3. Baseline characteristics were in general similar. Some differences exist, e.g., regarding mean LDL‐C, which is numerically lower in patients with prediction compared to patients without prediction.

### 3.3 Predictive Value of the ABC Model

**FIGURE 1:**
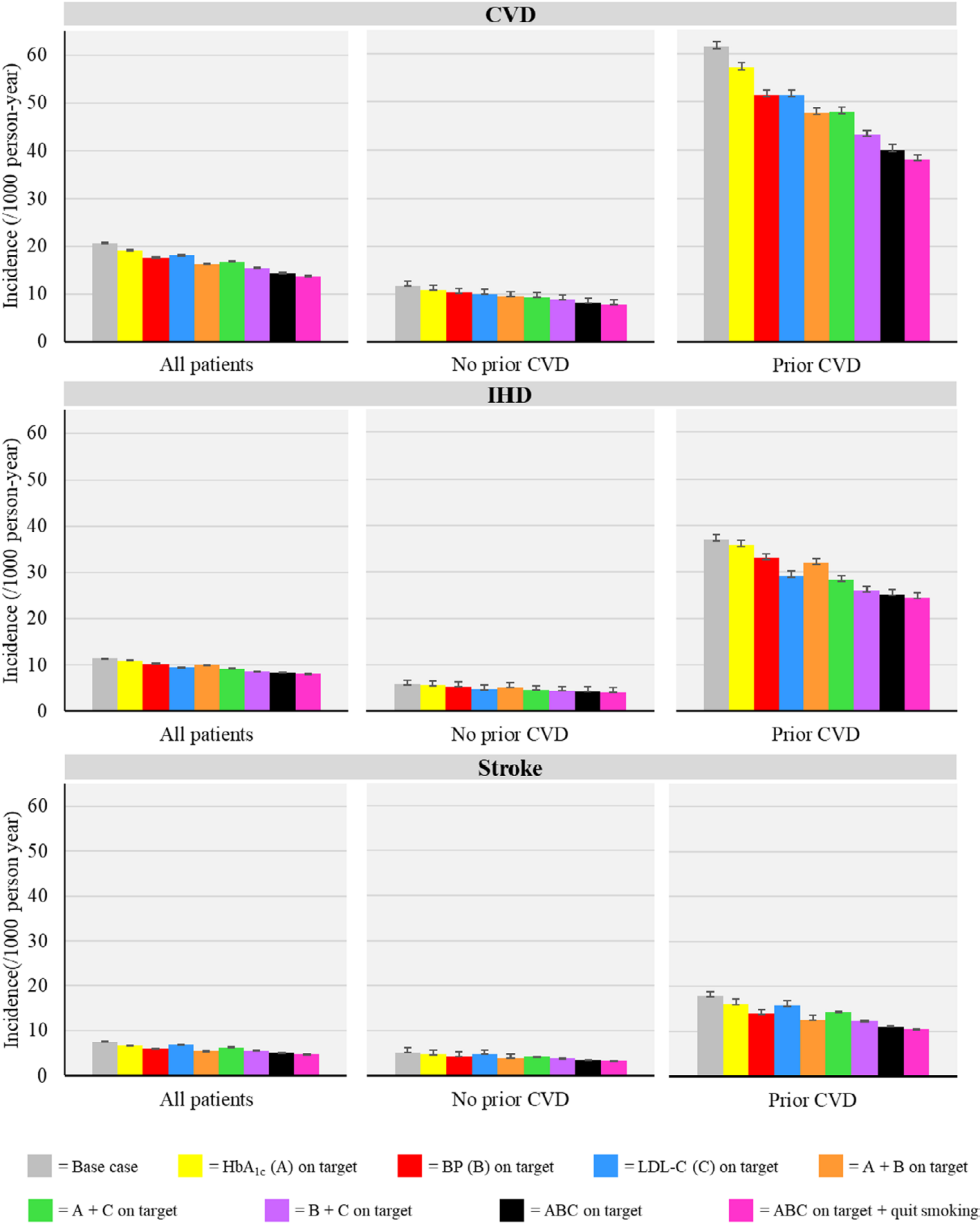
Predicted 3‐year incidence of CVD, IHD and stroke in patients with T2D with or without prior CVD with different scenarios of modelled achievement of ABC risk factor targets and smoking cessation. A on target: HbA1c < 7%; B on target: BP < 130/80 mmHg; C on target: LDL‐C < 2.6 mmol/L (if low/moderate CV risk) or LDL‐C < 1.8 mmol/L (if high CV risk) or LDL‐C < 1.4 mmol/L (if very high CV risk). Abbreviations: BP, blood pressure; CVD, cardiovascular disease; HbA1c, glycated haemoglobin A1c; IHD, ischemic heart disease; LDL‐C, low‐density lipoprotein‐cholesterol.

Most patients had simulated lower 3‐year CVD event rates in scenarios where single or multiple risk factor targets were achieved (Figure 1). The CVD group had the highest predicted 3‐year CVD incidence based on baseline risk factors with the largest simulated reduction in CVD incidence with control of single or multiple risk factors to target. Simulated event rates progressively declined with an increasing number of controlled risk factors with the largest simulated risk reduction for CVD, IHD and stroke in scenarios where all three ABC risk factor targets were achieved (Figure 1).

The estimated base incidence rates for CVD were 11.65 per 1000‐PY in the non‐CVD and 61.60 per 1000‐PY in the CVD group (Figure 1). Modelled achievement of all three ABC risk factor targets reduced the respective incidence rates to 8.13 per 1000‐PY and 40.01 per 1000‐PY. For IHD, the base incidence rate was 5.72 per 1000‐PY in the non‐CVD group and 37.04 per 1000‐PY in the CVD group, which were simulated to 4.19 per 1000‐PY and 25.14 per 1000‐PY, respectively, with achievement of all three ABC targets. For stroke, the base incidence rate was 5.2 per 1000‐PY in the non‐CVD and 17.85 per 1000‐PY in the CVD group with simulated incidence of 3.5 per 1000‐PY and 11.14 per 1000‐PY, respectively, with achievement of all three ABC targets (Figure 1).

**FIGURE 2:**
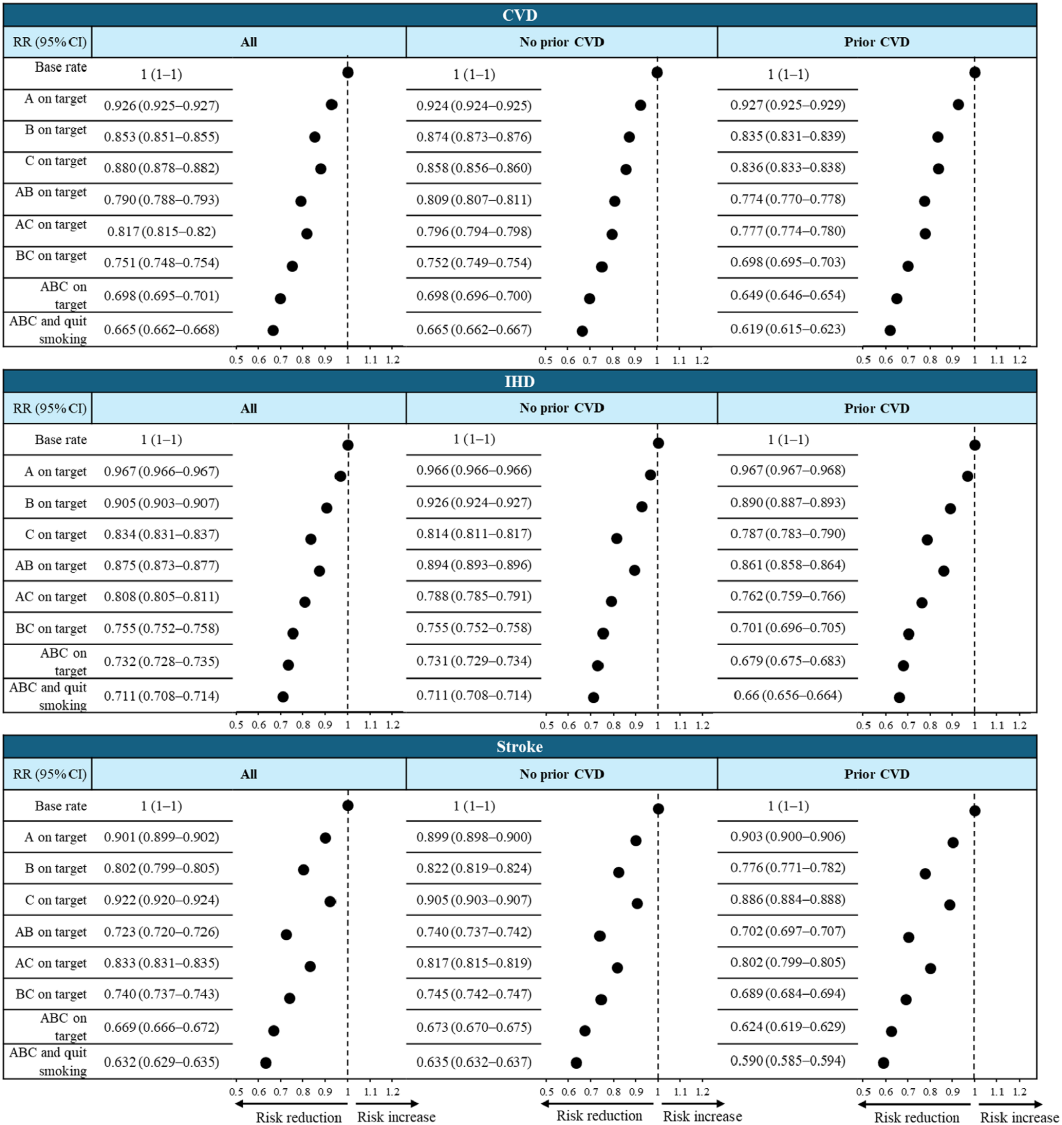
Relative risk prediction of 3‐year incidence of CVD, IHD and stroke in patients with T2D with or without prior CVD given different scenarios of modelled achievement of ABC risk factor targets and smoking cessation. The 95% CIs are not visually distinguishable on the forest plot due to narrow ranges. A on target: HbA_1c_ < 7%; B on target: BP < 130/80 mmHg; C on target: LDL‐C < 2.6 mmol/L (if low/moderate CV risk) or LDL‐C < 1.8 mmol/L (if high CV risk) or LDL‐C < 1.4 mmol/L (if very high CV risk). Abbreviations: BP, blood pressure; CI, confidence interval; CV, cardiovascular; CVD, cardiovascular disease; HbA_1c_, glycated haemoglobin A1c; IHD, ischaemic heart disease; LDL‐C, low‐density lipoprotein‐cholesterol; RR, relative risk.

Reduction of HbA_1c_ to < 7% reduced the simulated 3‐year incidence of CVD by 7.6% (non‐CVD group) and 7.3% (CVD group), IHD by 3.4% and 3.3% and stroke by 10.1% and 9.7%, respectively (Figure 2). Modelled adjustment of the BP to < 130/80 mmHg reduced CVD incidence by 12.6% and 16.5%, IHD by 7.4% and 11.0% and stroke by 17.8% and 22.4%, respectively. Modelled lowering of LDL‐C to risk‐based targets reduced CVD incidence by 14.2% and 16.4%, IHD by 18.6% and 21.3% and stroke by 9.5% and 11.4% in the respective groups.

Modelled achievement of all three ABC risk factor targets reduced the estimated 3‐year CVD incidence by 30.2% (non‐CVD group) and 35.1% (CVD group), IHD by 29.6% and 32.1% and stroke by 32.7% and 37.6%, respectively. When smoking cessation was added to the scenario, simulated CVD incidence decreased by 33.5% and 38.1%, IHD by 28.9% and 34.0% and stroke by 36.5% and 41.0% in the non‐CVD and CVD groups, respectively (Figure 2, Figure S1).

Table S4 shows the clinical profiles amongst patients with overweight/obesity. The base 3‐year simulated incidence of CVD was 21.17 per 1000‐PY, 20.67 per 1000‐PY and 19.12 per 1000‐PY in the 25–27.4 kg/m^2^, ≥ 27.5–29.9 kg/m^2^ and ≥ 30 kg/m^2^ groups, respectively. Modelled achievement of all three ABC risk factor targets reduced the 3‐year simulated incidence of CVD by 29.6%, 29.8% and 31.6% in these groups. Adding a 3% body weight reduction did not reduce CVD incidence with a similar reduction of 29.9%, 30.2% and 32.0%, respectively. With a 15% body weight reduction, the respective simulated risk reductions were 31.2%, 31.6% and 33.6%, with similar patterns for simulated IHD and stroke incidence (Figure S2).

### 3.4 Cost Analysis

**FIGURE 3:**
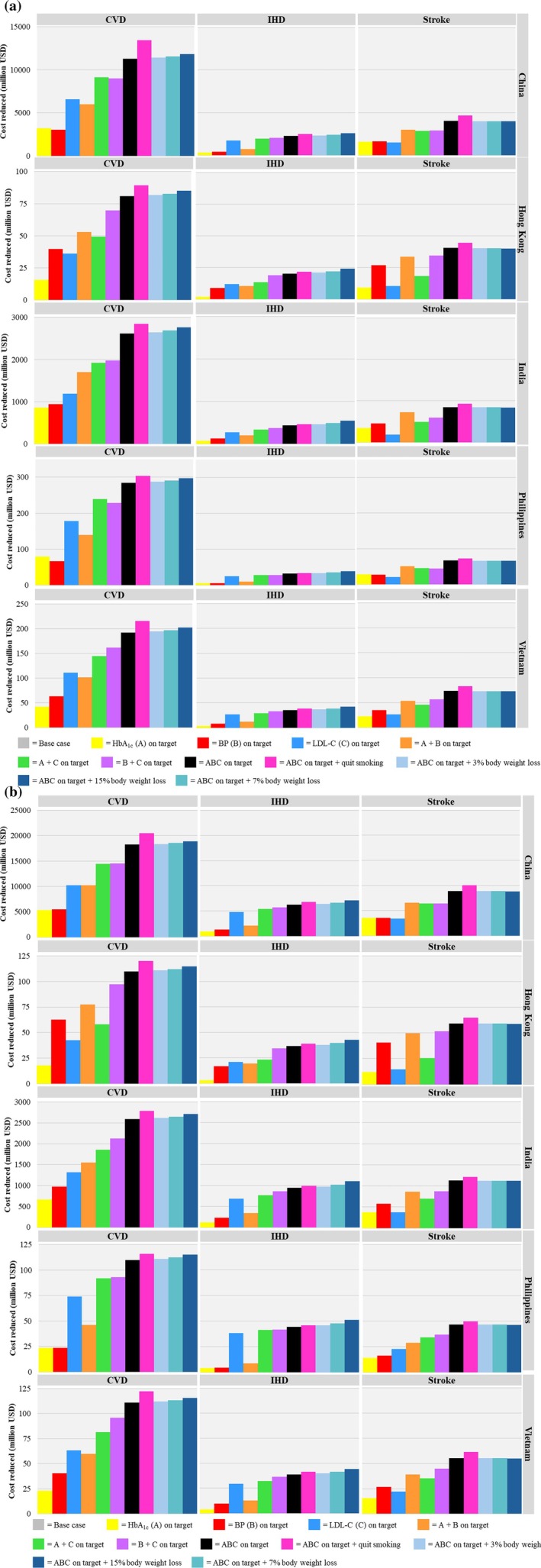
(A) Estimated cost reduction for different scenarios of modelled achievement of ABC risk factor targets, smoking cessation and body weight reduction in patients without prior CVD in five countries/jurisdictions. A on target: HbA_1c_ < 7%; B on target: BP < 130/80 mmHg; C on target: LDL‐C < 2.6 mmol/L (if low/moderate CV risk) or LDL‐C < 1.8 mmol/L (if high CV risk) or LDL‐C < 1.4 mmol/L (if very high CV risk). Abbreviations: BP, blood pressure; CV, cardiovascular; CVD, cardiovascular disease; HbA_1c_, glycated haemoglobin A1c; IHD, ischaemic heart disease; LDL‐C, low‐density lipoprotein‐cholesterol. (B) Estimated cost reduction for different scenarios of modelled achievement of ABC risk factor targets, smoking cessation and body weight reduction in patients with prior CVD in five countries/jurisdictions. A on target: HbA_1c_ < 7%; B on target: BP < 130/80 mmHg; C on target: LDL‐C < 2.6 mmol/L (if low/moderate CV risk) or LDL‐C < 1.8 mmol/L (if high CV risk) or LDL‐C < 1.4 mmol/L (if very high CV risk). Abbreviations: BP, blood pressure; CV, cardiovascular; CVD, cardiovascular disease HbA_1c_, glycated haemoglobin A1c; IHD, ischaemic heart disease; LDL‐C, low‐density lipoprotein‐cholesterol.

In the JADE Register, 89.5% of patients were from mainland China, Hong Kong, India, Philippines and Vietnam. The majority of patients did not have CVD with a baseline 3‐year simulated incidence of CVD ranging from 10.18 per 1000‐PY to 12.64 per 1000‐PY (Table S5). In the non‐CVD group, modelled achievement of HbA_1c_ target might avert 337 236 CVD events in mainland China (potential saving of US$ 3 233 761 739), 1192 events in Hong Kong (potential saving US$ 15 717 662), 188 583 events in India (potential saving US$ 859 494 998), 12 306 events in Philippines (potential saving US$ 79 582 479) and 7965 events in Vietnam (potential saving US$ 42 539 628; Figure 3A; Table S5). Achievement of LDL‐C or BP targets yielded similar estimated reductions and cost savings (Figure 3A,B; Table S5).

Simulation of scenario‐based achievement of all three ABC risk factors targets might avert 1 177 682 CVD events in mainland China (potential saving US$ 11 292 823 476), 6127 CVD events in Hong Kong (potential saving US$ 80 824 130), 575 390 events in India (potential saving US$ 2 622 429 194), 44 040 events in the Philippines (potential saving US$ 284 809 658), and 36 027 events in Vietnam (potential saving US$ 192 426 361) with further reduction in CVD events with addition of smoking cessation (Figure 3A,B; Table S5). Tables S6 and S7 show the cost analysis of simulated reduction in IHD and stroke events.

## 4 Discussion

Given the large number of individuals affected by diabetes with heterogeneous causes, trajectories and clinical outcomes [2], mathematical models such as the ABC model could simulate the impact of optimised intervention and its economic impact. Since each individual may have different combinations of risk factor control, mathematical modelling enables healthcare professionals (HCPs) to personalise therapeutic decisions and use these data to engage patients for motivating self‐management and treatment persistence [44]. Key cardiometabolic risk factors, namely HbA_1c_, BP, LDL‐C and smoking are inextricably linked, and their suboptimal control had amplifying effects on one another to accelerate organ damage. The application of the ABC model to Asian real‐world data gathered through the JADE Register is context relevant. By estimating the impact of risk factor reduction on CVD incidence, the ABC model demonstrates the value of multifactorial management of key risk factors on personal health and societal costs, even within a short period of 3 years.

In the JADE Register, only one‐third of patients with T2D had HbA_1c_ < 7% at baseline, similar to reports from most developing countries [21, 23, 45]. Therapeutic inertia, inadequate medical coverage, prohibitive treatment costs, insufficient access to education and support, fragmented care and treatment non‐adherence contributed to suboptimal glycemic control despite the proven efficacy of many oral glucose lowering drugs in clinical trial settings [46].

The benefits of multifactorial management for reducing CVD risk in patients with T2D have been proven [20]. In the latest BPROAD study, achieving a SBP target of 120 mmHg reduced CVD and related death by 20% [47]. The use of guideline‐directed medical therapy (GDMT) reduced CVD risk by 20%–40% in patients with or without T2D [5, 48]. Other real‐world evidence supported the substantial benefits of reducing HbA_1c_ and BP, using GDMT and integrated care to reduce CVD events [5, 37, 47, 49, 50]. Statin therapy is a cost‐effective medication recommended for all individuals with diabetes with age ≥ 40 years for reducing CVD risk [15, 20, 51, 52, 53], albeit its underutilization and low achievement of LDL‐C target are well documented [45, 54]. Likewise, low attainment of risk factor control and underutilization of GDMT such as renin angiotensin system inhibitors (RASi) are common [45, 55].

In this analysis, similar to the ICMR‐INDIAB study [22], less than 5% of patients achieved all three ABC risk factor targets with 44.4% of patients on statins and 40.9% of patients on RASi at baseline. In our simulated model, lowering LDL‐C and BP had the largest effect size for reducing 3‐year CVD risk. These estimations were similar to findings from large, multinational cohort analyses from the Global Cardiovascular Consortium [4, 56]. Although lowering HbA_1c_ alone had a smaller impact on 3‐year CVD risk than lowering BP or LDL‐C, the legacy benefits of lowering HbA_1c_ early on reducing all diabetes related endpoints in the long term have been proven by the UK Prospective Diabetes Study [57].

In high‐risk patients with moderate‐to‐severe obesity, glucagon‐like peptide‐1 (GLP‐1) and glucose‐dependent insulinotropic polypeptide receptor (GIPR) agonists reduced body weight, cardiometabolic risk factors and CVD risk [58, 59]. Intensive lifestyle intervention focusing on weight loss did not reduce CVD risk in overweight or obese adults with T2D, but the use of oral semaglutide, a GLP‐1R agonist, has shown cardio‐protective benefits [60, 61, 62]. In our analysis, simulated body weight reduction in Asians with T2D showed minimal incremental benefits. By contrast, simulated smoking cessation demonstrated to reduce CVD risk by 20% [63], averted more simulated CVD events than body weight reduction. In this context, simulations using the ABC model can inform public health policies, such as tobacco control and sugar‐sweetened beverage tax to complement individualised interventions [5].

The onset of diabetes‐related complications is accompanied by increased direct and indirect healthcare costs, loss of productivity and poor HRQoL [26, 41, 64]. Optimal control of ABC risk factors reduced healthcare costs based on observational and clinical trial data [20, 28, 46]. In mainland China, the combined direct and indirect costs of T2D in 2030 were projected to be US$ 47.2 billion [65], mainly attributed to treatment of complications [24, 46, 65]. The ABC model estimated the annual cost of CVD events in mainland China to be US$ 35.6 billion, 75% of the aforementioned total cost [65]. This close estimation supported the utility of the ABC model.

The estimated benefits and cost savings simulated by the ABC model depend on several assumptions. These include effective and sustained implementation of multifactorial risk factor management, encompassing access to GDMT, appropriate treatment initiation and intensification and a support system to enable persistence with medications and lifestyle modifications. To achieve these multiple goals, the healthcare system requires sufficient capacity and resources to deliver integrated diabetes care with equitable access. In this light, the heterogeneity of simulated CVD events amongst countries/jurisdictions is likely due to variations in ethnicity, demographics, risk factor distribution, time periods, quality of care, healthcare settings, access to care, education and medications.

### 4.1 Strengths and Limitations

The JADE Register is the first large‐scale web‐based quality improvement programme to establish a real‐world database for identifying care gaps and tracking clinical outcomes in Asian patients with diabetes. Participating sites included clinics and hospitals in private, public and subsidised sectors, which increased the generalizability of our results. Hong Kong and Sweden have universal medical coverage underpinning the high performance of the ABC model in European and Asian populations, adding validity to the study implications.

Some limitations of the ABC model should be acknowledged. By focusing mainly on HbA_1c_, BP and LDL‐C, the model simplifies the multifactorial nature of CVD risk in diabetes and may not capture additional biological, behavioural, or socioeconomic determinants although we have included smoking and body weight with strong behavioural components. Baseline characteristics of patients with and without prediction of 3‐year CVD incidence differed slightly. Since patients without prediction due to missing data had higher LDL‐C, there is a possibility that we might have underestimated the simulated event rates and potential number of events and costs averted in the cohort. The incidence of CVD and associated healthcare costs were only simulations which required validation of actual events. There are very few jurisdictions like Hong Kong [66] and Sweden [67] with universal health coverage and territory/nation‐wide databases that can track exposures, outcomes and healthcare costs on a population level. Short of this information in most countries, our results demonstrated the potential utility of using a validated outcome model to estimate the impact of interventions in different scenarios to inform practice and policies. Given the heterogeneity of clinical practice and patients' profiles, these models also enabled payors to prioritise context‐relevant interventions to create the largest impacts, although the cost estimations were scenario‐based and not actual costs. The crude estimates and imprecise classification of T1D and T2D in the IDF Atlas might introduce errors in our cost estimation. Although we used the World Bank PPP ratio to adjust for inter‐country cost differences, these estimates might be sensitive to assumptions regarding actual unit costs, resource utilisation, timelines used and discount rates. These simulations based on aggregated or secondary data sources have inherent precision and biases. We did not capture indirect costs and other intangible burdens which would underestimate the economic impact. These results may not be generalizable across different healthcare systems with variations in pricing structures, reimbursement policies, societal cultures and clinical practice. However, the rationale and methodology of these model‐based estimates are supported by a wealth of empirical evidence.

Using the 30‐year prospective HKDR data [68], we have developed and validated the Chinese Diabetes Outcome Model (CDOM) that predicted the lifetime risk of ten major endpoints. However, the incomplete documentation of albuminuria, a parameter in CDOM, due to lack of access or reimbursement in many enrollees of the JADE Register has limited the applicability of the more advanced CDOM to the cohort. Given the silent, progressive and heterogeneous nature of T2D, there is strong argument to create registers, improve quality of care, gather locally‐relevant real‐world evidence [69] and develop models for measuring disease burden and estimate the impact of intervention on outcomes and healthcare costs.

## 5 Conclusions

Our model‐based analysis highlights that multifactorial management of cardiometabolic risk factors may have a substantial impact on CVD prevention with reduced healthcare costs in patients with T2D. Our results iterate the value of comprehensive control of HbA_1c_, BP and LDL‐C and smoking cessation in the primary and secondary prevention of CVD. Using the ABC model to estimate CVD risk in a 3‐year horizon may support HCPs to individualise treatment plans including behavioural care whilst informing payors to improve care access for long‐term personal and societal benefits.

## Funding

This work was supported by Servier.

## Conflicts of Interest

The authors declare no conflicts of interest.

## Supporting information


**Table S1:** Prevalence of diabetes (type 1 and type 2) per country/jurisdiction.
**Table S2:** Cost of CVD estimated by the World Bank PPP ratio and exchange rate using the Hong Kong healthcare costs as a reference.
**Table S3:** Baseline characteristics of patients with and without prediction of 3‐year CVD incidence.
**Table S4:** Clinical profiles of patients with T2D in the JADE register, stratified by BMI categories.
**Table S5:** Reduction in the number of CVD events and associated healthcare costs with modelled achievement of ABC risk factor targets, smoking cessation and weight reduction in patients with T2D but without prior CVD in five countries/jurisdictions.
**Table S6:** Reduction in the number of IHD events and associated healthcare costs with modelled achievement of ABC risk factor targets, smoking cessation and weight reduction in patients with T2D but without prior CVD in five countries/Jurisdictions.
**Table S7:** Reduction in the number of stroke events and associated healthcare costs with modelled achievement of ABC risk factor targets, smoking cessation and weight reduction in patients with T2D but without prior CVD in five countries/jurisdictions.
**Figure S1:** Predicted 3‐year incidence of CVD comparing base case with simulated reduction to the target level of ABC risk factors, with and without smoking cessation.
**Figure S2:** Predicted 3‐year incidence of CVD in patients with T2D with different scenarios of modelled achievement of ABC risk factor targetss and body weight reduction.

## Data Availability

Individual‐level data cannot be shared but data supporting the findings of this study may be available from the corresponding author upon reasonable request.
