# Supplementary material for: Impact of Simulated Risk Factor Management on Cardiovascular Disease and Costs in Type 2 Diabetes Using the ABC Model
Source: Diabetes Obes Metab. 2026 Apr 13;28(7):5670–83. doi: 10.1111/dom.70755 (PMC13244001; doi:10.1111/dom.70755)
Supplement: Supplementary file 1 — Table S1: Prevalence of diabetes (type 1 and type 2) per country/jurisdiction. Table S2: Cost of CVD estimated by the World Bank PPP ratio and exchange rate using the Hong Kong healthcare costs as a reference. Table S3: Baseline characteristics of patients with and without prediction of 3‐year CVD incidence. Table S4: Clinical profiles of patients with T2D in the JADE register, stratified by BMI categories. Table S5: Reduction in the number of CVD events and associated healthcare costs with modelled achievement of ABC risk factor targets, smoking cessation and weight reduction in patients with T2D but without prior CVD in five countries/jurisdictions. Table S6: Reduction in the number of IHD events and associated healthcare costs with modelled achievement of ABC risk factor targets, smoking cessation and weight reduction in patients with T2D but without prior CVD in five countries/Jurisdictions. Table S7: Reduction in the number of stroke events and associated healthcare costs with modelled achievement of ABC risk factor targets, smoking cessation and weight reduction in patients with T2D but without prior CVD in five countries/jurisdictions. Figure S1: Predicted 3‐year incidence of CVD comparing base case with simulated reduction to the target level of ABC risk factors, with and without smoking cessation. Figure S2: Predicted 3‐year incidence of CVD in patients with T2D with different scenarios of modelled achievement of ABC risk factor targetss and body weight reduction. [file DOM-28-5670-s001.docx]

# **Supporting information captions**

**S1 Table. Prevalence of diabetes (type 1 and type 2) per country/jurisdiction.**

**S2 Table. Cost of CVD estimated by the World Bank PPP ratio and exchange rate using the Hong Kong healthcare costs as a reference.**

**S3 Table. Baseline characteristics of patients with and without prediction of 3-year CVD incidence.**

**S4 Table. Clinical profiles of patients with T2D in the JADE register, stratified by BMI categories.**

**S5 Table. Reduction in the number of CVD events and associated healthcare costs with modeled achievement of ABC risk factor targets, smoking cessation and weight reduction in patients with T2D without prior CVD in five countries/jurisdictions.**

**S6 Table. Reduction in the number of IHD events and associated healthcare costs with modeled achievement of ABC risk factor targets, smoking cessation, and weight reduction in patients with T2D but without prior CVD in five countries/Jurisdictions.**

**S7 Table. Reduction in the number of stroke events and associated healthcare costs with modeled achievement of ABC risk factor targets, smoking cessation, and weight reduction in patients with T2D but without prior CVD in five countries/jurisdictions.**

**S1 Figure. Predicted 3-year incidence of CVD comparing base case with simulated reduction to the target level of ABC risk factors, with and without smoking cessation.**

**S2 Figure. Predicted 3-year incidence of CVD in patients with T2D with different scenarios of modeled achievement of ABC risk factor targetss and body weight reduction.**

**S1 Table. Prevalence of Diabetes (Type 1 and Type 2) Per Country/Jurisdiction**

| **Country/jurisdiction** | **Number of people with diabetes^a^** | **Proportion with a prior history of CVD in the JADE Register** |
| --- | --- | --- |
| Mainland China | 140 869 600 | 21.74% |
| Hong Kong | 686 000 | 20.76% |
| India | 74 194 700 | 15.34% |
| Philippines | 4 303 900 | 8.27% |
| Vietnam | 3 994 100 | 9.52% |

^a^Number of people with diabetes per country was obtained from the IDF Diabetes Atlas 2021.

CVD, cardiovascular disease; IDF, International Diabetes Federation; JADE Register, Joint Asia Diabetes Evaluation Register.

**S2 Table. Cost of CVD Estimated by the World Bank PPP Ratio and Exchange Rate Using the Hong Kong healthcare Costs as a Reference**

|  | **China** | **Hong Kong** | **India** | **Philippines** | **Vietnam** |
| --- | --- | --- | --- | --- | --- |
| PPP conversion factor | 3.6370 | 5.5297 | 20.2026 | 19.2623 | 6802.4707 |
| PPP to exchange rate | 0.5134 | 0.7063 | 0.2440 | 0.3463 | 55.6304 |
| Exchange rate | 7.0840 | 7.8296 | 82.7897 | 0.2860 | 23787.3192 |
| **Cost of complications (US$)** | | | | | |
| CVD | 9589.02 | 13190.85 | 4557.66 | 6467.08 | 5341.13 |
| IHD | 5706.68 | 7850.23 | 2712.38 | 3848.73 | 3178.65 |
| Stroke | 13675.74 | 18812.62 | 6500.07 | 9223.27 | 7617.45 |

Note: World Bank Purchasing Power Parity ratios were adapted from the Cost-of-Living Index,^1^ and Hong Kong healthcare costs were estimated from Lui, et al. 2024.^2^
CVD, cardiovascular disease; IHD, ischemic heart disease; PPP, purchasing power parity; US$, United States Dollar.

**S3 Table. Baseline Characteristics of Patients with and without Prediction of 3-year CVD Incidence**

|  | **Number of patients with data** | **Patients with prediction** | **Number of patients with data** | **Patients without prediction^a^** | **Standardized mean difference** |
| --- | --- | --- | --- | --- | --- |
| Total number of persons, n |  | 75 420 |  | 28 538 |  |
| Mean age, years (SD) | 75 420 | 58 (11.7) | 28 538 | 56.5 (11.7) | 0.123 |
| Male sex, n (%) | 75 420 | 41 403 (54.9) | 28 531 | 14 059 (49.3) | 0.113 |
| Median disease duration, years (IQR) | 75 420 | 6 (2–13) | 26 663 | 5 (2–11) | 0.158 |
| Non-smoker, n (%) | 75 420 | 54 365 (72.1) | 26 691 | 19 637 (73.6) | 0.040 |
| Ex-smoker, n (%) |  | 11 988 (15.9) |  | 3864 (14.5) |  |
| Current smoker, n (%) |  | 9067 (12.0) |  | 3190 (12.0) |  |
| Prior IHD, n (%) | 75 420 | 7832 (10.4) | 28 538 | 2125 (7.4) | 0.103 |
| Prior stroke, n (%) | 75 420 | 3140 (4.2) | 28 538 | 818 (2.9) | 0.070 |
| Mean BW, kg (SD) (male) | 41 403 | 73.3 (13.1) | 12 772 | 72.5 (14.2) | 0.055 |
| Mean BW, kg (SD) (female) | 34 017 | 63.2 (12.5) | 12 803 | 63.2 (14.6) | 0.001 |
| Mean Waist, cm (SD) (male) | 38 386 | 92.7 (11.2) | 8479 | 90.3 (11.4) | 0.215 |
| Mean Waist, cm (SD) (female) | 31 188 | 89 (11.8) | 7235 | 86 (13.3) | 0.243 |
| Mean BMI, kg/m^2^ (SD) | 75 420 | 26.3 (4.54) | 22 738 | 25.9 (4.52) | 0.077 |
| Mean HbA_1c_, (SD) | 75 420 | 7.92 (1.82) | 14 670 | 8.19 (2.08) | 0.137 |
| Mean SBP, mmHg (SD) | 75 420 | 132 (17.5) | 26 186 | 130 (17.1) | 0.127 |
| Mean DBP, mmHg (SD) | 75 420 | 78.3 (9.83) | 26 008 | 79.9 (9.18) | 0.168 |
| Mean LDL-C, mmol/L (SD) | 75 420 | 2.58 (0.937) | 7847 | 2.84 (1.07) | 0.259 |
| Mean HDL-C, mmol/L (SD) | 74 581 | 1.19 (0.359) | 9291 | 1.19 (0.562) | 0.008 |
| Median TG, mmol/L (IQR) | 74 781 | 1.5 (1.1–2.1) | 13 921 | 1.76 (1.27–2.37) | 0.199 |
| HbA_1c_ <7%, n (%) | 75 420 | 26 635 (35.3) | 14 670 | 4591 (31.3) | 0.085 |
| BP <130/80 mmHg, n (%) | 75 420 | 22 600 (30.0) | 26 037 | 5967 (22.9) | 0.160 |
| LDL-C <CV-risk based target, n (%) | 68 956 | 17 092 (24.8) | 2475 | 329 (13.3) | 0.296 |
| LDL-C <2.6 mmol/L (low-moderate CV risk), n (%) | 24 352 | 12 432 (51.1) | 362 | 155 (42.8) | 0.166 |
| LDL-C <1.8 mmol/L (high CV-risk), n (%) | 16 879 | 2105 (12.5) | 313 | 49 (15.7) | 0.092 |
| LDL-C <1.4 mmol/L (very high CV-risk), n (%) | 27 725 | 2555 (9.2) | 1800 | 125 (6.9) | 0.083 |
| On 0 target, n (%) | 68 956 | 25 747 (37.3) | 1383 | 587 (42.4) | 0.166 |
| On 1 target, n (%) |  | 26 985 (39.1) |  | 559 (40.4) |  |
| On 2 targets, n (%) |  | 13 162 (19.1) |  | 205 (14.8) |  |
| On all 3 targets, n (%) |  | 3062 (4.4) |  | 32 (2.3) |  |
| On OGLD only, n (%) | 75 420 | 51 833 (68.7) | 28 538 | 18 948 (66.4) | 0.050 |
| On HbA_1c_ target whilst on OGLD only, n (%) | 51 833 | 20 934 (40.4) | 9214 | 3177 (34.5) | 0.122 |
| On insulin ± OGLD, n (%) | 75 420 | 16 052 (21.3) | 28 538 | 4927 (17.3) | 0.102 |
| On HbA_1c_ target whilst on insulin, n (%) | 16 052 | 2620 (16.3) | 2984 | 521 (17.5) | 0.030 |
| On statin, n (%) | 64 378 | 31 450 (48.9) | 24 020 | 7774 (32.4) | 0.341 |
| On LDL-C target whilst on statin, n (%) | 28 985 | 7672 (26.5) | 998 | 114 (11.4) | 0.391 |
| On RASi, n (%) | 66 136 | 28 976 (43.8) | 23 264 | 7545 (32.4) | 0.236 |
| On BP target whilst on RASi, n (%) | 28 976 | 7146 (24.7) | 7054 | 1177 (16.7) | 0.198 |
| On AGI, n (%) | 67 165 | 4484 (6.7) | 23 218 | 1106 (4.8) | 0.082 |
| On DPP4i, n (%) | 67 165 | 13 563 (20.2) | 23 218 | 3814 (16.4) | 0.098 |
| On glinides, n (%) | 67 165 | 984 (1.5) | 23 218 | 165 (0.7) | 0.073 |
| On metformin, n (%) | 67 165 | 47 004 (70.0) | 23 218 | 13 417 (57.8) | 0.256 |
| On SGLT2i, n (%) | 67 165 | 386 (0.6) | 23 218 | 22 (0.1) | 0.083 |
| On sulphonylurea, n (%) | 67 165 | 31 034 (46.2) | 23 218 | 9603 (41.4) | 0.098 |
| On thiazolidinedione, n (%) | 67 165 | 4280 (6.4) | 23 218 | 1855 (8.0) | 0.063 |
| On GLP1-RA, n (%) | 67 165 | 243 (0.4) | 23 219 | 54 (0.2) | 0.024 |

^a^Due to missing age, sex, duration of diabetes, smoking, BMI, HbA1c, SBP, DBP, LDL-C or history of CVD.
AGI, alpha-glucosidase inhibitor; CVD, cardiovascular disease; BMI, body-mass index; BP, blood pressure; BW, body weight; IHD, ischemic heart disease; CV, cardiovascular; CVD, cardiovascular disease;
DBP, diastolic blood pressure; DPP4i, dipeptidyl peptidase-4 inhibitor; GLP1-RA, glucagon-like peptide-1 receptor agonist; HbA_1c_, glycated haemoglobin A1c; HDL-C; high-density lipoprotein-cholesterol;
IQR, interquartile range; LDL-C, low-density lipoprotein-cholesterol; OGLD, oral glucose lowering drug; RASi, renin angiotensin system inhibitors; SBP, systolic blood pressure; SD, standard deviation; SGLT2i, sodium-glucose co-transporter 2 inhibitor; TG, triglyceride.

**S4 Table. Clinical Profiles of Patients With T2D in the JADE Register, Stratified by BMI Categories**

|  | **Number of persons evaluated** | **BMI <18.5** | **Number of persons evaluated** | **BMI  18.5–<23** | **Number of persons evaluated** | **BMI 23–<25** | **Number of persons evaluated** | **BMI 25–<27.5** | **Number of persons evaluated** | **BMI 27.5–<30** | **Number of persons evaluated** | **BMI ≥30** |
| --- | --- | --- | --- | --- | --- | --- | --- | --- | --- | --- | --- | --- |
| Total number of patients, n | - | 1682 | - | 20 515 | - | 20 430 | - | 23 878 | - | 15 612 | - | 16 041 |
| Mean age, years (SD) | 1682 | 59.0 (13.3) | 20 515 | 59.9 (11.8) | 20 430 | 58.5 (11.5) | 23 878 | 57.4 (11.4) | 15 612 | 56.4 (11.5) | 16 041 | 54.4 (11.6) |
| Male sex, n (%) | 1682 | 778 (46.3) | 20 513 | 10 218 (49.8) | 20 429 | 11 211 (54.9) | 23 876 | 14 010 (58.7) | 15 612 | 8943 (57.3) | 16 040 | 7751 (48.3) |
| Median disease duration, Years, (IQR) | 1668 | 5 (1–12) | 20 296 | 7 (2–13) | 20 212 | 6 (2–13) | 23 588 | 6 (2–12) | 15 397 | 6 (2–12) | 15 832 | 6 (2–12) |
| Non-smoker, n (%) | 1670 | 1213 (72.6) | 20 317 | 14 768 (72.7) | 20 235 | 14 451 (71.4) | 23 655 | 16 396 (69.3) | 15 456 | 11 050 (71.5) | 15 888 | 12 317 (77.5) |
| Ex-smoker, n (%) | 1670 | 256 (15.3) | 20 317 | 3076 (15.1) | 20 235 | 3367 (16.6) | 23 655 | 4,065 (17.2) | 15 456 | 2379 (15.4) | 15 888 | 1982 (12.5) |
| Current smoker, n (%) | 1670 | 201 (12.0) | 20 317 | 2473 (12.2) | 20 235 | 2417 (11.9) | 23 655 | 3,194 (13.5) | 15 456 | 2027 (13.1) | 15 888 | 1589 (10.0) |
| Prior IHD, n (%) | 1682 | 100 (5.9) | 20 515 | 1744 (8.5) | 20 430 | 2193 (10.7) | 23 878 | 2,630 (11.0) | 15 612 | 1613 (10.3) | 16 041 | 1466 (9.1) |
| Prior stroke, n (%) | 1682 | 69 (4.1) | 20 515 | 843 (4.1) | 20 430 | 812 (4.0) | 23 878 | 860 (3.6) | 15 612 | 595 (3.8) | 16 041 | 550 (3.4) |
| Mean BW, kg (SD) (male) | 778 | 47.8 (5.36) | 10 218 | 59.7 (5.85) | 11 211 | 67.3 (5.48) | 14 010 | 73.6 (5.97) | 8943 | 79.8 (6.62) | 7751 | 92.7 (13) |
| Mean BW, kg (SD) (female) | 904 | 41.9 (4.82) | 10 295 | 51.4 (4.99) | 9218 | 58.1 (4.67) | 9866 | 63.1 (5.32) | 6669 | 68.6 (5.81) | 8289 | 80.6 (11.9) |
| Mean Waist, cm (SD) (male) | 598 | 75.2 (9.24) | 8837 | 83.0 (7.53) | 9926 | 88.2 (7.44) | 12 501 | 92.2 (7.6) | 7945 | 96.9 (8.44) | 6762 | 106.0 (11.2) |
| Mean Waist, cm (SD) (female) | 662 | 71.6 (9.54) | 8481 | 79.3 (8.09) | 7801 | 83.6 (8.72) | 8413 | 88.6 (8.71) | 5674 | 93.7 (8.55) | 7107 | 102.0 (11.1) |
| Mean BMI, kg/m^2^ (SD) | 1682 | 17.2 (1.2) | 20 515 | 21.4 (1.16) | 20 430 | 24.0 (0.579) | 23 878 | 26.2 (0.711) | 15 612 | 28.6 (0.709) | 16 041 | 33.7 (4.09) |
| Mean HbA_1c_, (SD) | 1389 | 8.3 (2.47) | 18 133 | 7.9 (2.01) | 17 960 | 7.9 (1.85) | 21 046 | 7.9 (1.76) | 13 926 | 8.0 (1.74) | 14 648 | 8.1 (1.79) |
| Mean SBP, mmHg (SD) | 1662 | 125.0 (20.2) | 20 333 | 129.0 (18.3) | 20 275 | 131.0 (17.4) | 23 725 | 132.0 (16.8) | 15 510 | 133.0 (16.7) | 15 944 | 134.0 (16.9) |
| Mean DBP, mmHg (SD) | 1660 | 74.4 (10.7) | 20 305 | 76.4 (9.98) | 20 231 | 78.2 (9.44) | 23 682 | 79.0 (9.26) | 15 478 | 79.9 (9.37) | 15 921 | 81.1 (9.71) |
| Mean LDL-C, mmol/L (SD) | 1250 | 2.6 (0.993) | 16 689 | 2.6 (0.948) | 16 647 | 2.6 (0.951) | 19 563 | 2.6 (0.939) | 13 133 | 2.6 (0.927) | 13 603 | 2.6 (0.946) |
| Mean HDL-C, mmol/L(SD) | 1247 | 1.4 (0.553) | 16 782 | 1.3 (0.412) | 16 743 | 1.2 (0.356) | 19 677 | 1.2 (0.334) | 13 248 | 1.1 (0.336) | 13 756 | 1.1 (0.349) |
| Median TG, mmol/L (IQR) | 1343 | 1.1 (0.791–1.58) | 17 622 | 1.3  (0.9–1.91) | 17 794 | 1.5  (1.1–2.1) | 20 867 | 1.6  (1.16–2.2) | 13 863 | 1.7  (1.2–2.24) | 14 296 | 1.7  (1.22–2.26) |
| HbA_1c_ <7%, n (%) | 1389 | 521 (37.5) | 18 133 | 7191 (39.7) | 17 960 | 6548 (36.5) | 21 046 | 7174 (34.1) | 13 926 | 4346 (31.2) | 14 648 | 4279 (29.2) |
| BP <130/80 mmHg, n (%) | 1661 | 731 (44.0) | 20 310 | 7446 (36.7) | 20 235 | 6244 (30.9) | 23 688 | 6342 (26.8) | 15 483 | 3578 (23.1) | 15 925 | 3283 (20.6) |
| LDL-C <CV-risk based target, n (%) | 1076 | 314 (29.2) | 14 687 | 3494 (23.8) | 14 521 | 3352 (23.1) | 16 902 | 4075 (24.1) | 11 233 | 2722 (24.2) | 11 849 | 3236 (27.3) |
| LDL-C <2.6 mmol/L (low-moderate CV-risk), n (%) | 399 | 239 (59.9) | 4872 | 2492 (51.1) | 4742 | 2371 (50.0) | 5709 | 2926 (51.3) | 3954 | 1976 (50.0) | 4737 | 2452 (51.8) |
| LDL-C <1.8 mmol/L (high CV-risk), n (%) | 249 | 41 (16.5) | 3722 | 487 (13.1) | 3719 | 488 (13.1) | 4284 | 496 (11.6) | 2620 | 304 (11.6) | 2328 | 293 (12.6) |
| LDL-C <1.4 mmol/L (very high CV-risk), n (%) | 428 | 34 (7.9) | 6093 | 515 (8.5) | 6060 | 493 (8.1) | 6909 | 653 (9.5) | 4659 | 442 (9.5) | 4784 | 491 (10.3) |
| On 0 target, n (%) | 1060 | 284 (26.8) | 14 507 | 4556 (31.4) | 14 278 | 5225 (36.6) | 16 673 | 6350 (38.1) | 11 065 | 4513 (40.8) | 11 729 | 4994 (42.6) |
| On 1 target, n (%) | 1060 | 407 (38.4) | 14 507 | 5692 (39.2) | 14 278 | 5554 (38.9) | 16 673 | 6547 (39.3) | 11 065 | 4395 (39.7) | 11 729 | 4524 (38.6) |
| On 2 targets, n (%) | 1060 | 272 (25.7) | 14 507 | 3363 (23.2) | 14 278 | 2806 (19.7) | 16 673 | 3094 (18.6) | 11 065 | 1792 (16.2) | 11 729 | 1879 (16.0) |
| On all 3 targets, n (%) | 1060 | 97 (9.2) | 14 507 | 896 (6.2) | 14 278 | 693 (4.9) | 16 673 | 682 (4.1) | 11 065 | 365 (3.3) | 11 729 | 332 (2.8) |
| On OGLD only, n (%) | 1682 | 1036 (61.6) | 20 515 | 14 046 (68.5) | 20, 430 | 14 115 (69.1) | 23 878 | 16 580 (69.4) | 15 612 | 10 823 (69.3) | 16 041 | 10 936 (68.2) |
| On HbA_1c_ target whilst on OGLD only, n (%) | 854 | 371 (43.4) | 12 366 | 5579 (45.1) | 12 316 | 5125 (41.6) | 14 496 | 5632 (38.9) | 9530 | 3397 (35.6) | 9931 | 3355 (33.8) |
| On insulin ± OGLD, n (%) | 1682 | 399 (23.7) | 20 515 | 4025 (19.6) | 20 430 | 4116 (20.1) | 23 878 | 4842 (20.3) | 15 612 | 3248 (20.8) | 16 041 | 3588 (22.4) |
| On HbA_1c_ target whilst on insulin, n (%) | 338 | 64 (18.9) | 3642 | 703 (19.3) | 3745 | 679 (18.1) | 4472 | 760 (17.0) | 3034 | 429 (14.1) | 3379 | 435 (12.9) |
| On statin, n (%) | 1512 | 392 (25.9) | 17 729 | 7086 (40.0) | 17 411 | 7870 (45.2) | 20 256 | 9709 (47.9) | 13 127 | 6512 (49.6) | 13 539 | 6575 (48.6) |
| On LDL-C target whilst on statin, n (%) | 293 | 86 (29.4) | 5554 | 1426 (25.7) | 6105 | 1491 (24.4) | 7375 | 1900 (25.8) | 5012 | 1296 (25.9) | 5279 | 1520 (28.8) |
| On RASi, n (%) | 1502 | 376 (25.0) | 18 092 | 5969 (33.0) | 17 786 | 7056 (39.7) | 20 603 | 8874 (43.1) | 13 286 | 6254 (47.1) | 13 592 | 6983 (51.4) |
| On BP target whilst on RASi, n (%) | 372 | 123 (33.1) | 5927 | 1664 (28.1) | 7019 | 1729 (24.6) | 8825 | 2037 (23.1) | 6228 | 1305 (21.0) | 6946 | 1339 (19.3) |
| On AGI, n (%) | 1470 | 85 (5.8) | 17 871 | 1235 (6.9) | 17 809 | 1190 (6.7) | 20 907 | 1328 (6.4) | 13 572 | 809 (6.0) | 14 041 | 846 (6.0) |
| On DPP4i, n (%) | 1470 | 185 (12.6) | 17 871 | 2737 (15.3) | 17 809 | 3070 (17.2) | 20 907 | 4045 (19.3) | 13 572 | 3094 (22.8) | 14 041 | 3514 (25.0) |
| On glinides, n (%) | 1470 | 20 (1.4) | 17 871 | 302 (1.7) | 17 809 | 261 (1.5) | 20 907 | 269 (1.3) | 13 572 | 148 (1.1) | 14 041 | 126 (0.9) |
| On metformin, n (%) | 1470 | 729 (53.9) | 17 871 | 11 310 (63.3) | 17 809 | 11 817 (66.4) | 20 907 | 14 399 (68.9) | 13 572 | 9651 (71.1) | 14 041 | 10 389 (74.0) |
| On SGLT2i, n (%) | 1470 | 5 (0.3) | 17 871 | 64 (0.4) | 17 809 | 68 (0.4) | 20 907 | 92 (0.4) | 13 572 | 61 (0.4) | 14 041 | 117 (0.8) |
| On sulphonylurea, n (%) | 1470 | 563 (38.3) | 17 871 | 7926 (44.4) | 17 809 | 8346 (46.9) | 20 907 | 9688 (46.3) | 13 572 | 6410 (47.2) | 14 041 | 6444 (45.9) |
| On thiazolidinedione, n (%) | 1470 | 68 (4.6) | 17 871 | 948 (5.3) | 17 809 | 1124 (6.3) | 20 907 | 1617 (7.7) | 13 572 | 1044 (7.7) | 14 041 | 1078 (7.7) |
| On GLP1-RA, n (%) | 1470 | 0 (0.0) | 17 871 | 4 (0.0) | 17 810 | 24 (0.1) | 20 907 | 35 (0.2) | 13 572 | 54 (0.4) | 14 041 | 171 (1.2) |

Note: BMI categories were determined using the cut-off points detailed in the WHO Expert Consultation 2004.^38^ LDL-C <2.6 mmol/L (if low/moderate CV risk) or LDL-C <1.8 mmol/L (if high CV risk) or LDL-C <1.4 mmol/L (if very high CV risk). AGI, alpha-glucosidase inhibitor; CVD, cardiovascular disease; BMI, body-mass index; BP, blood pressure; BW, body weight; IHD, ischemic heart disease; DBP, diastolic blood pressure; DPP4i, dipeptidyl peptidase-4 inhibitor; GLP1-RA, glucagon-like peptide-1 receptor agonist; HbA_1c_, glycated haemoglobin A1c; HDL-C; high-density lipoprotein-cholesterol; LDL-C, low-density lipoprotein-cholesterol; OGLD, oral glucose lowering drug; RASi, renin angiotensin system inhibitors; SBP, systolic blood pressure; SD, standard deviation; SGLT2i, sodium-glucose co-transporter 2 inhibitor; T2D, type 2 diabetes; TG, triglyceride.

**S5 Table. Reduction in the Number of CVD Events and Associated Healthcare Costs With Modeled Achievement of ABC Risk Factor Targets, Smoking Cessation and Weight Reduction in Patients With T2D Without Prior CVD in Five Countries/Jurisdictions**

| **Country/**  **jurisdictions** | **Scenario** | **Incidence  (per 1000 PY)** | **Number of events** | **Reduction in the number of events** | **Cost**  **(US$)** | **Cost reduction (US$)** | **Percentage difference** |
| --- | --- | --- | --- | --- | --- | --- | --- |
| **China (N = 110 244 549)** | Base rate | 11.42 | 3 713 976 | 0 | 35 613 401 019 | 0 | 0.0% |
|  | A on target | 10.37 | 3 376 740 | 337 236 | 32 379 639 280 | 3 233 761 739 | 9.1% |
|  | B on target | 10.43 | 3 395 362 | 318 614 | 32 558 203 015 | 3 055 198 004 | 8.6% |
|  | C on target | 9.28 | 3 028 358 | 685 619 | 29 038 989 040 | 6 574 411 980 | 18.5% |
|  | AB on target | 9.48 | 3 090 326 | 623 650 | 29 633 208 152 | 5 980 192 867 | 16.8% |
|  | AC on target | 8.46 | 2 762 823 | 951 153 | 26 492 774 905 | 9 120 626 114 | 25.6% |
|  | BC on target | 8.51 | 2 777 553 | 936 423 | 26 634 022 565 | 8 979 378 454 | 25.2% |
|  | ABC on target | 7.76 | 2 536 294 | 1 177 682 | 24 320 577 544 | 11 292 823 476 | 31.7% |
|  | ABC on target and quit smoking | 7.06 | 2 311 752 | 1 402 224 | 22 167 444 367 | 13 445 956 652 | 37.8% |
|  | ABC on target + 3% BW loss | 7.72 | 2 525 010 | 1 188 966 | 24 212 374 909 | 11 401 026 110 | 32.0% |
|  | ABC on target + 5% BW loss | 7.70 | 2 517 515 | 1 196 461 | 24 140 510 248 | 11 472 890 771 | 32.2% |
|  | ABC on target + 7% BW loss | 7.68 | 2 510 043 | 1 203 933 | 24 068 861 168 | 11 544 539 852 | 32.4% |
|  | ABC on target + 10% BW loss | 7.64 | 2 498 877 | 1 215 099 | 23 961 790 338 | 11 651 610 681 | 32.7% |
|  | ABC on target + 15% BW loss | 7.59 | 2 480 379 | 1 233 597 | 23 784 407 668 | 11 828 993 351 | 33.2% |
| **Hong Kong (N = 543 586)** | Base rate | 12.64 | 20 220 | 0 | 266 717 521 | 0 | 0.0% |
|  | A on target | 11.88 | 19 028 | 1192 | 250 999 858 | 15 717 662 | 5.9% |
|  | B on target | 10.72 | 17 202 | 3018 | 226 905 345 | 39 812 175 | 14.9% |
|  | C on target | 10.89 | 17 471 | 2749 | 230 455 978 | 36 261 543 | 13.6% |
|  | AB on target | 10.08 | 16 199 | 4021 | 213 673 925 | 53 043 595 | 19.9% |
|  | AC on target | 10.26 | 16 478 | 3741 | 217 365 173 | 49 352 348 | 18.5% |
|  | BC on target | 9.29 | 14 932 | 5288 | 196 966 736 | 69 750 785 | 26.2% |
|  | ABC on target | 8.76 | 14 093 | 6127 | 185 893 390 | 80 824 130 | 30.3% |
|  | ABC on target and quit smoking | 8.35 | 13 448 | 6772 | 177 388 426 | 89 329 094 | 33.5% |
|  | ABC on target + 3% BW loss | 8.72 | 14 028 | 6191 | 185 046 587 | 81 670 933 | 30.6% |
|  | ABC on target + 5% BW loss | 8.69 | 13 986 | 6234 | 184 484 228 | 82 233 293 | 30.8% |
|  | ABC on target + 7% BW loss | 8.66 | 13 943 | 6277 | 183 923 603 | 82 793 918 | 31.0% |
|  | ABC on target + 10% BW loss | 8.62 | 13 880 | 6340 | 183 085 906 | 83 631 614 | 31.4% |
|  | ABC on target + 15% BW loss | 8.56 | 13 775 | 6445 | 181 698 342 | 85 019 178 | 31.9% |
| **India (N = 62 813 233)** | Base rate | 10.18 | 1 889 318 | 0 | 8 610 866 825 | 0 | 0.0% |
|  | A on target | 9.15 | 1 700 736 | 188 583 | 7 751 371 827 | 859 494 998 | 10.0% |
|  | B on target | 9.05 | 1 682 167 | 207 151 | 7 666 742 804 | 944 124 020 | 11.0% |
|  | C on target | 8.75 | 1 627 820 | 261 498 | 7 419 048 436 | 1 191 818 389 | 13.8% |
|  | AB on target | 8.14 | 1 515 990 | 373 328 | 6 909 363 062 | 1 701 503 763 | 19.8% |
|  | AC on target | 7.88 | 1 467 107 | 422 211 | 6 686 571 750 | 1 924 295 074 | 22.3% |
|  | BC on target | 7.82 | 1 456 017 | 433 301 | 6 636 027 548 | 1 974 839 277 | 22.9% |
|  | ABC on target | 7.05 | 1 313 929 | 575 390 | 5 988 437 631 | 2 622 429 194 | 30.5% |
|  | ABC on target and quit smoking | 6.77 | 1 263 633 | 625 685 | 5 759 206 182 | 2 851 660 643 | 33.1% |
|  | ABC on target + 3% BW loss | 7.01 | 1 307 592 | 581 726 | 5 959 558 479 | 2 651 308 346 | 30.8% |
|  | ABC on target + 5% BW loss | 6.99 | 1 303 385 | 585 933 | 5 940 384 666 | 2 670 482 158 | 31.0% |
|  | ABC on target + 7% BW loss | 6.97 | 1 299 192 | 590 126 | 5 921 273 773 | 2 689 593 051 | 31.2% |
|  | ABC on target + 10% BW loss | 6.93 | 1 292 928 | 596 390 | 5 892 724 949 | 2 718 141 876 | 31.6% |
|  | ABC on target + 15% BW loss | 6.88 | 1 282 557 | 606 761 | 5 845 455 212 | 2 765 411 613 | 32.1% |
| **Philippines (N = 3 947 968)** | Base rate | 10.96 | 127 734 | 0 | 826 063 551 | 0 | 0.0% |
|  | A on target | 9.89 | 115 428 | 12 306 | 746 481 072 | 79 582 479 | 9.6% |
|  | B on target | 10.07 | 117 482 | 10 251 | 759 767 924 | 66 295 627 | 8.0% |
|  | C on target | 8.57 | 100 190 | 27 544 | 647 935 514 | 178 128 037 | 21.6% |
|  | AB on target | 9.09 | 106 199 | 21 534 | 686 799 611 | 139 263 940 | 16.9% |
|  | AC on target | 7.75 | 90 739 | 36 995 | 586 815 334 | 239 248 217 | 29.0% |
|  | BC on target | 7.90 | 92 446 | 35 288 | 597 854 720 | 228 208 831 | 27.6% |
|  | ABC on target | 7.14 | 83 694 | 44 040 | 541 253 893 | 284 809 658 | 34.5% |
|  | ABC on target and quit smoking | 6.89 | 80 822 | 46 912 | 522 682 347 | 303 381 204 | 36.7% |
|  | ABC on target + 3% BW loss | 7.11 | 83 312 | 44 422 | 538 784 692 | 287 278 859 | 34.8% |
|  | ABC on target + 5% BW loss | 7.09 | 83 058 | 44 675 | 537 144 994 | 288 918 557 | 35.0% |
|  | ABC on target + 7% BW loss | 7.07 | 82 806 | 44 928 | 535 510 425 | 290 553 126 | 35.2% |
|  | ABC on target + 10% BW loss | 7.03 | 82 428 | 45 306 | 533 068 153 | 292 995 398 | 35.5% |
|  | ABC on target + 15% BW loss | 6.98 | 81 802 | 45 931 | 529 023 114 | 297 040 437 | 36.0% |
| **Vietnam (N = 3 613 862)** | Base rate | 11.58 | 123 374 | 0 | 658 954 127 | 0 | 0.0% |
|  | A on target | 10.82 | 115 409 | 7965 | 616 414 499 | 42 539 628 | 6.5% |
|  | B on target | 10.45 | 111 528 | 11 846 | 595 685 120 | 63 269 007 | 9.6% |
|  | C on target | 9.60 | 102 642 | 20 732 | 548 223 255 | 110 730 872 | 16.8% |
|  | AB on target | 9.77 | 104 343 | 19 030 | 557 309 961 | 101 644 165 | 15.4% |
|  | AC on target | 9.00 | 96 284 | 27 090 | 514 263 122 | 144 691 005 | 22.0% |
|  | BC on target | 8.70 | 93 105 | 30 268 | 497 287 424 | 161 666 703 | 24.5% |
|  | ABC on target | 8.16 | 87 346 | 36 027 | 466 527 765 | 192 426 361 | 29.2% |
|  | ABC on target and quit smoking | 7.74 | 82 936 | 40 437 | 442 973 059 | 215 981 068 | 32.8% |
|  | ABC on target + 3% BW loss | 8.12 | 86 971 | 36 402 | 464 525 939 | 194 428 187 | 29.5% |
|  | ABC on target + 5% BW loss | 8.10 | 86 723 | 36 651 | 463 196 200 | 195 757 927 | 29.7% |
|  | ABC on target + 7% BW loss | 8.07 | 86 474 | 36 899 | 461 870 296 | 197 083 830 | 29.9% |
|  | ABC on target + 10% BW loss | 8.04 | 86 103 | 37 270 | 459 888 609 | 199 065 518 | 30.2% |
|  | ABC on target + 15% BW loss | 7.98 | 85 488 | 37 885 | 456 604 821 | 202 349 306 | 30.7% |

Note: A on target: HbA_1c_ <7%; B on target: BP <130/80 mmHg; C on target: LDL-C <2.6 mmol/L (if low/moderate CV risk) or LDL-C <1.8 mmol/L (if high CV risk) or LDL-C <1.4 mmol/L (if very high CV risk).
CVD, cardiovascular disease; BW, body weight; HbA_1c_, glycated haemoglobin A1c; LDL-C, low-density lipoprotein-cholesterol; PY, person years; T2D, type 2 diabetes; US$, United States Dollar.

**S6 Table. Reduction in the Number of IHD Events and Associated Healthcare Costs With Modeled Achievement of ABC Risk Factor Targets, Smoking Cessation, and Weight Reduction in Patients With T2D but Without Prior CVD in Five Countries/Jurisdictions**

| **Country/ jurisdiction** | **Scenario** | **Incidence  (per 1000 PY)** | **Number of events** | **Reduction in the number of events** | **Cost**  **(US$)** | **Cost reduction (US$)** | **Percentage difference** |
| --- | --- | --- | --- | --- | --- | --- | --- |
| **China (N = 110 244 549)** | Base rate | 5.47 | 1 794 409 | 0 | 10 240 121 249 | 0 | 0.0% |
|  | A on target | 5.25 | 1 723 315 | 71 093 | 9 834 413 354 | 405 707 894 | 4.0% |
|  | B on target | 5.25 | 1 722 074 | 72 335 | 9 827 330 405 | 412 790 844 | 4.0% |
|  | C on target | 4.18 | 1 373 760 | 420 649 | 7 839 611 110 | 2 400 510 139 | 23.4% |
|  | AB on target | 5.04 | 1 654 331 | 140 078 | 9 440 742 177 | 799 379 072 | 7.8% |
|  | AC on target | 4.02 | 1 322 141 | 472 268 | 7 545 037 669 | 2 695 083 580 | 26.3% |
|  | BC on target | 4.02 | 1 322 417 | 471 992 | 7 546 612 906 | 2 693 508 343 | 26.3% |
|  | ABC on target | 3.87 | 1 273 061 | 521 348 | 7 264 954 097 | 2 975 167 152 | 29.1% |
|  | ABC on target and quit smoking | 3.66 | 1 204 451 | 589 958 | 6 873 417 054 | 3 366 704 195 | 32.9% |
|  | ABC on target + 3% BW loss | 3.81 | 1 253 536 | 540 873 | 7 153 529 625 | 3 086 591 624 | 30.1% |
|  | ABC on target + 5% BW loss | 3.77 | 1 240 689 | 553 720 | 7 080 216 036 | 3 159 905 212 | 30.9% |
|  | ABC on target + 7% BW loss | 3.73 | 1 227 976 | 566 433 | 7 007 668 275 | 3 232 452 974 | 31.6% |
|  | ABC on target + 10% BW loss | 3.68 | 1 209 155 | 585 254 | 6 900 264 648 | 3 339 856 601 | 32.6% |
|  | ABC on target + 15% BW loss | 3.58 | 1 178 438 | 615 971 | 6 724 972 888 | 3 515 148 361 | 34.3% |
| **Hong Kong (N = 543 586)** | Base rate | 6.11 | 9878 | 0 | 77 545 002 | 0 | 0.0% |
|  | A on target | 5.95 | 9615 | 263 | 75 483 631 | 2 061 372 | 2.7% |
|  | B on target | 5.53 | 8936 | 942 | 70 146 247 | 7 398 755 | 9.5% |
|  | C on target | 5.03 | 8135 | 1743 | 63 859 757 | 13 685 245 | 17.6% |
|  | AB on target | 5.38 | 8700 | 1178 | 68 298 303 | 9 246 699 | 11.9% |
|  | AC on target | 4.90 | 7930 | 1948 | 62 252 465 | 15 292 538 | 19.7% |
|  | BC on target | 4.57 | 7398 | 2480 | 58 073 252 | 19 471 750 | 25.1% |
|  | ABC on target | 4.45 | 7213 | 2665 | 56 624 364 | 20 920 639 | 27.0% |
|  | ABC on target and quit smoking | 4.33 | 7018 | 2860 | 55 093 124 | 22 451 878 | 29.0% |
|  | ABC on target + 3% BW loss | 4.38 | 7099 | 2779 | 55 731 943 | 21 813 060 | 28.1% |
|  | ABC on target + 5% BW loss | 4.34 | 7025 | 2853 | 55 144 999 | 22 400 003 | 28.9% |
|  | ABC on target + 7% BW loss | 4.29 | 6951 | 2927 | 54 564 377 | 22 980 625 | 29.6% |
|  | ABC on target + 10% BW loss | 4.22 | 6841 | 3037 | 53 705 144 | 23 839 858 | 30.7% |
|  | ABC on target + 15% BW loss | 4.11 | 6663 | 3215 | 52 303 722 | 25 241 281 | 32.6% |
| **India (N = 62 813 233)** | Base rate | 5.17 | 966 672 | 0 | 2 621 985 064 | 0 | 0.0% |
|  | A on target | 4.93 | 922 902 | 43 769 | 2 503 265 615 | 118 719 449 | 4.5% |
|  | B on target | 4.86 | 909 569 | 57 102 | 2 467 101 394 | 154 883 670 | 5.9% |
|  | C on target | 4.24 | 793 672 | 173 000 | 2 152 743 372 | 469 241 691 | 17.9% |
|  | AB on target | 4.64 | 868 653 | 98 019 | 2 356 121 243 | 265 863 821 | 10.1% |
|  | AC on target | 4.05 | 758 670 | 208 002 | 2 057 804 059 | 564 181 005 | 21.5% |
|  | BC on target | 4.00 | 749 594 | 217 078 | 2 033 185 978 | 588 799 086 | 22.5% |
|  | ABC on target | 3.83 | 716 788 | 249 884 | 1 944 204 609 | 677 780 455 | 25.8% |
|  | ABC on target and quit smoking | 3.74 | 701 226 | 265 446 | 1 901 994 034 | 719 991 029 | 27.5% |
|  | ABC on target + 3% BW loss | 3.76 | 704 857 | 261 815 | 1 911 843 604 | 710 141 460 | 27.1% |
|  | ABC on target + 5% BW loss | 3.72 | 697 017 | 269 655 | 1 890 577 390 | 731 407 674 | 27.9% |
|  | ABC on target + 7% BW loss | 3.68 | 689 266 | 277 406 | 1 869 554 035 | 752 431 028 | 28.7% |
|  | ABC on target + 10% BW loss | 3.62 | 677 805 | 288 867 | 1 838 468 136 | 783 516 928 | 29.9% |
|  | ABC on target + 15% BW loss | 3.52 | 659 137 | 307 535 | 1 787 832 697 | 834 152 366 | 31.8% |
| **Philippines (N = 3 947 968)** | Base rate | 5.58 | 65 517 | 0 | 252 156 902 | 0 | 0.0% |
|  | A on target | 5.33 | 62 676 | 2841 | 241 221 731 | 10 935 171 | 4.3% |
|  | B on target | 5.33 | 62 678 | 2839 | 241 230 850 | 10 926 052 | 4.3% |
|  | C on target | 3.98 | 46 903 | 18 614 | 180 517 418 | 71 639 483 | 28.4% |
|  | AB on target | 5.10 | 59 963 | 5554 | 230 782 581 | 21 374 321 | 8.5% |
|  | AC on target | 3.82 | 44 950 | 20 567 | 172 999 869 | 79 157 033 | 31.4% |
|  | BC on target | 3.83 | 45 053 | 20 464 | 173 397 683 | 78 759 219 | 31.2% |
|  | ABC on target | 3.66 | 43 167 | 22 350 | 166 137 519 | 86 019 383 | 34.1% |
|  | ABC on target and quit smoking | 3.59 | 42 289 | 23 228 | 162 757 926 | 89 398 976 | 35.5% |
|  | ABC on target + 3% BW loss | 3.61 | 42 482 | 23 034 | 163 503 789 | 88 653 113 | 35.2% |
|  | ABC on target + 5% BW loss | 3.57 | 42 033 | 23 484 | 161 772 075 | 90 384 827 | 35.8% |
|  | ABC on target + 7% BW loss | 3.53 | 41 588 | 23 929 | 160 059 386 | 92 097 515 | 36.5% |
|  | ABC on target + 10% BW loss | 3.47 | 40 929 | 24 588 | 157 525 549 | 94 631 353 | 37.5% |
|  | ABC on target + 15% BW loss | 3.38 | 39 856 | 25 661 | 153 394 554 | 98 762 348 | 39.2% |
| **Vietnam (N = 3 613 862)** | Base rate | 5.50 | 59 189 | 0 | 188 142 482 | 0 | 0.0% |
|  | A on target | 5.34 | 57 485 | 1705 | 182 723 196 | 5 419 287 | 2.9% |
|  | B on target | 5.18 | 55 687 | 3502 | 177 010 095 | 11 132 387 | 5.9% |
|  | C on target | 4.30 | 46 314 | 12 876 | 147 214 672 | 40 927 811 | 21.8% |
|  | AB on target | 5.03 | 54 089 | 5100 | 171 931 273 | 16 211 209 | 8.6% |
|  | AC on target | 4.18 | 45 060 | 14 129 | 143 230 937 | 44 911 546 | 23.9% |
|  | BC on target | 4.06 | 43 720 | 15 469 | 138 971 403 | 49 171 079 | 26.1% |
|  | ABC on target | 3.95 | 42 541 | 16 648 | 135 223 008 | 52 919 475 | 28.1% |
|  | ABC on target and quit smoking | 3.82 | 41 215 | 17 974 | 131 008 719 | 57 133 763 | 30.4% |
|  | ABC on target + 3% BW loss | 3.89 | 41 912 | 17 277 | 133 223 777 | 54 918 706 | 29.2% |
|  | ABC on target + 5% BW loss | 3.85 | 41 498 | 17 691 | 131 907 648 | 56 234 835 | 29.9% |
|  | ABC on target + 7% BW loss | 3.81 | 41 088 | 18 101 | 130 604 714 | 57 537 769 | 30.6% |
|  | ABC on target + 10% BW loss | 3.75 | 40 481 | 18 708 | 128 674 761 | 59 467 722 | 31.6% |
|  | ABC on target + 15% BW loss | 3.66 | 39 489 | 19 700 | 125 522 268 | 62 620 214 | 33.3% |

Note: A on target: HbA_1c_ <7%; B on target: BP <130/80 mmHg; C on target: LDL-C <2.6 mmol/L (if low/moderate CV risk) or LDL-C <1.8 mmol/L (if high CV risk) or LDL-C <1.4 mmol/L (if very high CV risk).
BW, body weight; HbA_1c_, glycated haemoglobin A1c; IHD, ischemic heart disease; LDL-C, low-density lipoprotein-cholesterol; PY, person years; T2D, type 2 diabetes; US$, United States Dollar.

**S7 Table. Reduction in the Number of Stroke Events and Associated Healthcare Costs With Modeled Achievement of ABC Risk Factor Targets, Smoking Cessation, and Weight Reduction in Patients With T2D but Without Prior CVD in Five Countries/Jurisdictions**

| **Country/ jurisdiction** | **Scenario** | **Incidence  (per 1000 PY)** | **Number of events** | **Reduction in the number of events** | **Cost**  **(US$)** | **Cost reduction (US$)** | **Percentage difference** |
| --- | --- | --- | --- | --- | --- | --- | --- |
| **China (N = 110 244 549)** | Base rate | 5.24 | 1 720 514 | 0 | 23 529 309 621 | 0 | 0.0% |
|  | A on target | 4.57 | 1 499 996 | 220 518 | 20 513 561 021 | 3 015 748 600 | 12.8% |
|  | B on target | 4.53 | 1 488 842 | 231 672 | 20 361 022 044 | 3 168 287 577 | 13.5% |
|  | C on target | 4.54 | 1 491 957 | 228 558 | 20 403 614 450 | 3 125 695 171 | 13.3% |
|  | AB on target | 3.96 | 1 300 965 | 419 550 | 17 791 656 623 | 5 737 652 998 | 24.4% |
|  | AC on target | 3.97 | 1 304 776 | 415 738 | 17 843 779 725 | 5 685 529 896 | 24.2% |
|  | BC on target | 3.94 | 1 295 069 | 425 445 | 17 711 031 751 | 5 818 277 870 | 24.7% |
|  | ABC on target | 3.45 | 1 134 744 | 585 770 | 15 518 471 120 | 8 010 838 502 | 34.0% |
|  | ABC on target and quit smoking | 3.09 | 1 018 415 | 702 099 | 13 927 582 104 | 9 601 727 518 | 40.8% |
|  | ABC on target + 3% BW loss | 3.45 | 1 135 907 | 584 607 | 15 534 369 867 | 7 994 939 754 | 34.0% |
|  | ABC on target + 5% BW loss | 3.45 | 1 136 683 | 583 832 | 15 544 978 230 | 7 984 331 391 | 33.9% |
|  | ABC on target + 7% BW loss | 3.46 | 1 137 459 | 583 055 | 15 555 593 957 | 7 973 715 664 | 33.9% |
|  | ABC on target + 10% BW loss | 3.46 | 1 138 624 | 581 890 | 15 571 531 367 | 7 957 778 255 | 33.8% |
|  | ABC on target + 15% BW loss | 3.47 | 1 140 569 | 579 945 | 15 598 130 611 | 7 931 179 011 | 33.7% |
| **Hong Kong (N = 543 586)** | Base rate | 5.76 | 9304 | 0 | 175 041 834 | 0 | 0.0% |
|  | A on target | 5.29 | 8566 | 738 | 161 149 135 | 13 892 699 | 7.9% |
|  | B on target | 4.55 | 7367 | 1938 | 138 592 343 | 36 449 491 | 20.8% |
|  | C on target | 5.20 | 8416 | 888 | 158 332 473 | 16 709 361 | 9.5% |
|  | AB on target | 4.19 | 6792 | 2513 | 127 770 061 | 47 271 773 | 27.0% |
|  | AC on target | 4.80 | 7764 | 1541 | 146 055 097 | 28 986 737 | 16.6% |
|  | BC on target | 4.13 | 6690 | 2615 | 125 851 891 | 49 189 943 | 28.1% |
|  | ABC on target | 3.81 | 6179 | 3125 | 116 245 660 | 58 796 174 | 33.6% |
|  | ABC on target and quit smoking | 3.60 | 5842 | 3462 | 109 911 459 | 65 130 375 | 37.2% |
|  | ABC on target + 3% BW loss | 3.82 | 6186 | 3119 | 116 367 416 | 58 674 418 | 33.5% |
|  | ABC on target + 5% BW loss | 3.82 | 6190 | 3115 | 116 448 659 | 58 593 175 | 33.5% |
|  | ABC on target + 7% BW loss | 3.82 | 6194 | 3110 | 116 529 959 | 58 511 874 | 33.4% |
|  | ABC on target + 10% BW loss | 3.82 | 6201 | 3104 | 116 652 019 | 58 389 814 | 33.4% |
|  | ABC on target + 15% BW loss | 3.83 | 6212 | 3093 | 116 855 743 | 58 186 091 | 33.2% |
| **India (N = 62 813 233)** | Base rate | 4.26 | 797 943 | 0 | 5 186 690 544 | 0 | 0.0% |
|  | A on target | 3.68 | 689 437 | 108 506 | 4 481 392 071 | 705 298 474 | 13.6% |
|  | B on target | 3.56 | 667 688 | 130 255 | 4 340 025 175 | 846 665 369 | 16.3% |
|  | C on target | 3.85 | 721 657 | 76 286 | 4 690 826 135 | 495 864 409 | 9.6% |
|  | AB on target | 3.08 | 578 497 | 219 447 | 3 760 271 573 | 1 426 418 972 | 27.5% |
|  | AC on target | 3.33 | 623 588 | 174 355 | 4 053 370 115 | 1 133 320 429 | 21.9% |
|  | BC on target | 3.23 | 606 237 | 191 707 | 3 940 582 760 | 1 246 107 784 | 24.0% |
|  | ABC on target | 2.80 | 525 391 | 272 552 | 3 415 083 816 | 1 771 606 729 | 34.2% |
|  | ABC on target and quit smoking | 2.67 | 500 525 | 297 418 | 3 253 450 558 | 1 933 239 986 | 37.3% |
|  | ABC on target + 3% BW loss | 2.80 | 525 975 | 271 968 | 3 418 879 375 | 1 767 811 169 | 34.1% |
|  | ABC on target + 5% BW loss | 2.81 | 526 365 | 271 578 | 3 421 412 152 | 1 765 278 392 | 34.0% |
|  | ABC on target + 7% BW loss | 2.81 | 526 755 | 271 188 | 3 423 946 854 | 1 762 743 690 | 34.0% |
|  | ABC on target + 10% BW loss | 2.81 | 527 340 | 270 603 | 3 427 752 520 | 1 758 938 024 | 33.9% |
|  | ABC on target + 15% BW loss | 2.82 | 528 318 | 269 625 | 3 434 104 943 | 1 752 585 601 | 33.8% |
| **Philippines (N = 3 947 968)** | Base rate | 4.97 | 58 441 | 0 | 539 016 244 | 0 | 0.0% |
|  | A on target | 4.33 | 50 923 | 7518 | 469 678 144 | 69 338 099 | 12.9% |
|  | B on target | 4.37 | 51 478 | 6963 | 474 797 489 | 64 218 755 | 11.9% |
|  | C on target | 4.29 | 50 430 | 8011 | 465 126 109 | 73 890 135 | 13.7% |
|  | AB on target | 3.81 | 44 900 | 13 541 | 414 124 056 | 124 892 188 | 23.2% |
|  | AC on target | 3.73 | 43 975 | 14 466 | 405 595 560 | 133 420 684 | 24.8% |
|  | BC on target | 3.77 | 44 427 | 14 014 | 409 763 498 | 129 252 746 | 24.0% |
|  | ABC on target | 3.29 | 38 739 | 19 702 | 357 295 870 | 181 720 374 | 33.7% |
|  | ABC on target and quit smoking | 3.15 | 37 188 | 21 253 | 342 998 561 | 196 017 682 | 36.4% |
|  | ABC on target + 3% BW loss | 3.29 | 38 779 | 19 662 | 357 667 987 | 181 348 257 | 33.6% |
|  | ABC on target + 5% BW loss | 3.29 | 38 806 | 19 635 | 357 916 287 | 181 099 957 | 33.6% |
|  | ABC on target + 7% BW loss | 3.29 | 38 833 | 19 608 | 358 164 766 | 180 851 478 | 33.6% |
|  | ABC on target + 10% BW loss | 3.30 | 38 873 | 19 568 | 358 537 817 | 180 478 426 | 33.5% |
|  | ABC on target + 15% BW loss | 3.30 | 38 941 | 19 500 | 359 160 463 | 179 855 781 | 33.4% |
| **Vietnam (N = 3 613 862)** | Base rate | 5.46 | 58 725 | 0 | 447 333 200 | 0 | 0.0% |
|  | A on target | 4.97 | 53 523 | 5202 | 407 710 932 | 39 622 269 | 8.9% |
|  | B on target | 4.74 | 50 993 | 7732 | 388 438 524 | 58 894 676 | 13.2% |
|  | C on target | 4.83 | 51 942 | 6783 | 395 667 218 | 51 665 982 | 11.5% |
|  | AB on target | 4.31 | 46 479 | 12 246 | 354 049 847 | 93 283 353 | 20.9% |
|  | AC on target | 4.41 | 47 446 | 11 279 | 361 417 988 | 85 915 212 | 19.2% |
|  | BC on target | 4.20 | 45 259 | 13 465 | 344 760 506 | 102 572 694 | 22.9% |
|  | ABC on target | 3.83 | 41 340 | 17 385 | 314 904 226 | 132 428 975 | 29.6% |
|  | ABC on target and quit smoking | 3.62 | 39 007 | 19 718 | 297 131 907 | 150 201 293 | 33.6% |
|  | ABC on target + 3% BW loss | 3.84 | 41 381 | 17 344 | 315 215 138 | 132 118 062 | 29.5% |
|  | ABC on target + 5% BW loss | 3.84 | 41 408 | 17 317 | 315 422 587 | 131 910 614 | 29.5% |
|  | ABC on target + 7% BW loss | 3.84 | 41 435 | 17 290 | 315 630 174 | 131 703 027 | 29.4% |
|  | ABC on target + 10% BW loss | 3.85 | 41 476 | 17 249 | 315 941 814 | 131 391 387 | 29.4% |
|  | ABC on target + 15% BW loss | 3.85 | 41 544 | 17 180 | 316 461 908 | 130 871 293 | 29.3% |

Note: A on target: HbA_1c_ <7%; B on target: BP <130/80 mmHg; C on target: LDL-C <2.6 mmol/L (if low/moderate CV risk) or LDL-C <1.8 mmol/L (if high CV risk) or LDL-C <1.4 mmol/L (if very high CV risk). BW, body weight; HbA_1c_, glycated haemoglobin A1c; LDL-C, low-density lipoprotein-cholesterol; PY, person years; T2D, type 2 diabetes; US$, United States Dollar.

**S1 Figure. Predicted 3-Year Incidence of CVD Comparing Base Case With Simulated Reduction to the Target Level of ABC Risk Factors, With and Without Smoking Cessation**


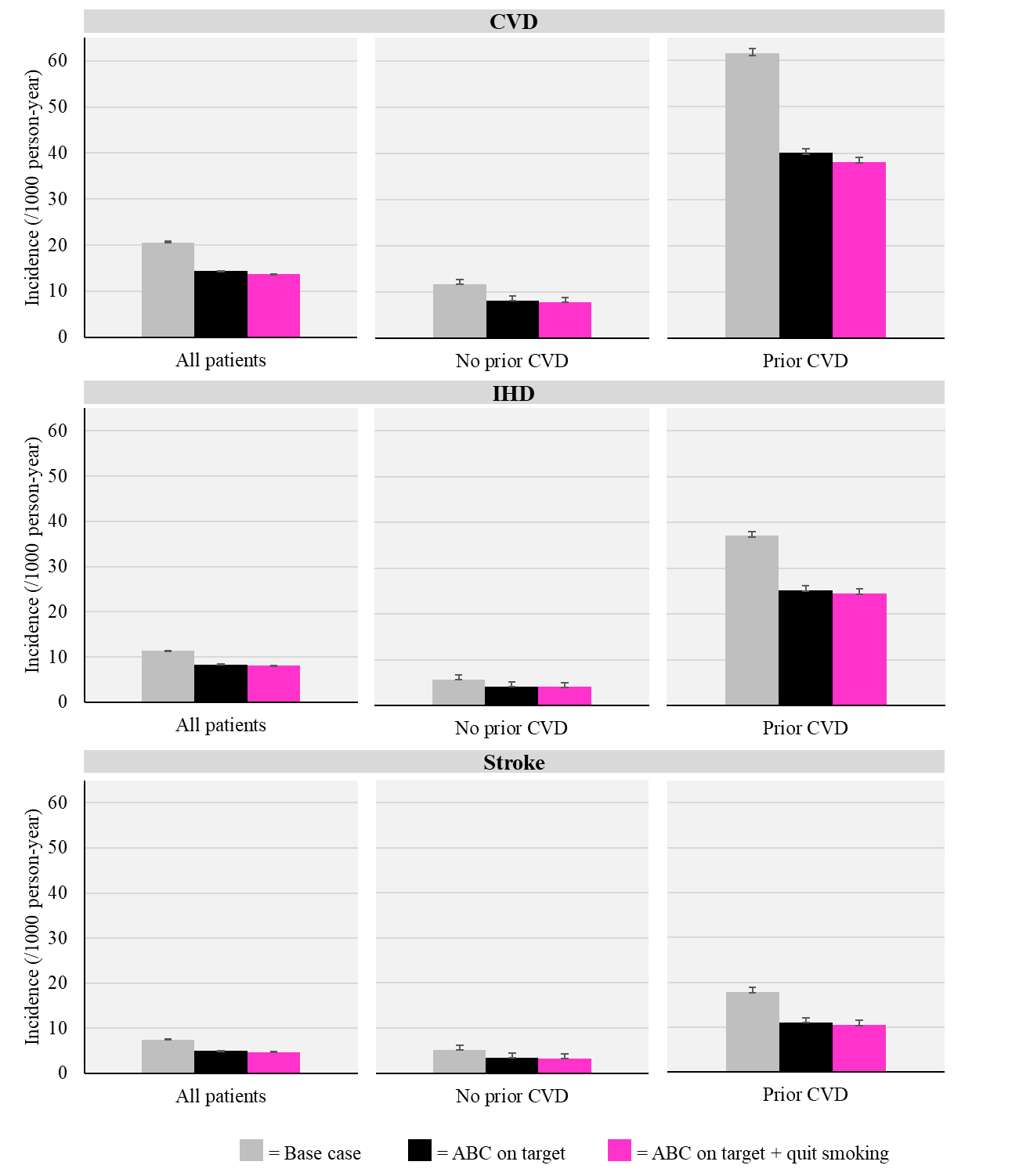


Note: error bars represent 95% confidence intervals. A on target: HbA_1c_ <7%; B on target: BP <130/80 mmHg; C on target: LDL-C <2.6 mmol/L (if low/moderate CV risk) or LDL-C <1.8 mmol/L (if high CV risk) or LDL-C <1.4 mmol/L (if very high CV risk).
CVD, cardiovascular disease; IHD, ischemic heart disease; HbA_1c_, glycated haemoglobin A1c;
LDL-C, low-density lipoprotein-cholesterol.

**S2 Figure. Predicted 3-Year Incidence of CVD in Patients With T2D With Different Scenarios of Modeled Achievement of ABC Risk Factor Targets and Body Weight Reduction**


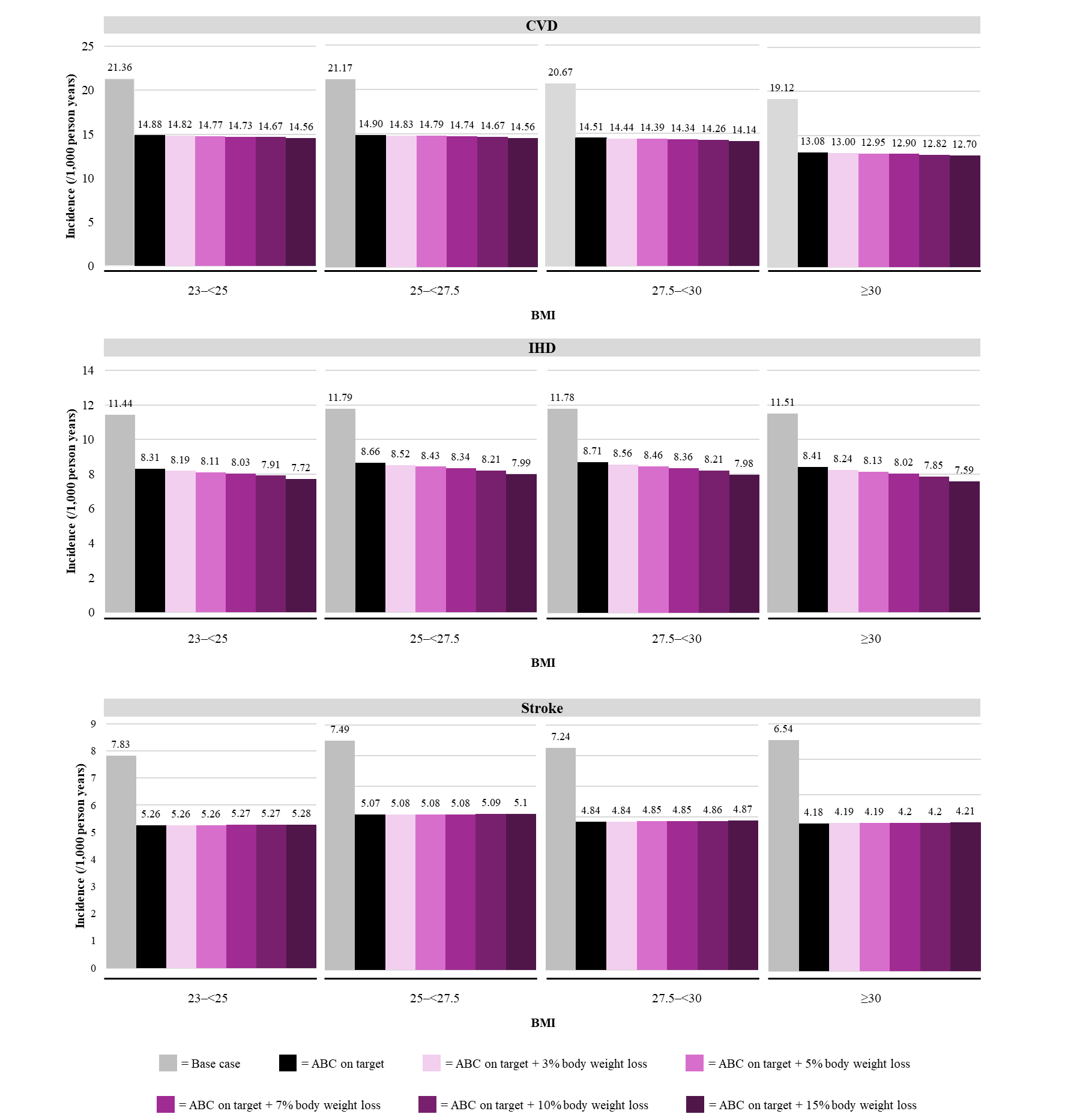


Note: BMI categories were determined using the cut-off points detailed in the WHO Expert Consultation 2004.^3^ A on target: HbA_1c_ <7%; B on target: BP <130/80 mmHg; C on target: LDL-C <2.6 mmol/L (if low/moderate CV risk) or LDL-C <1.8 mmol/L (if high CV risk) or LDL-C <1.4 mmol/L (if very high CV risk). CVD, cardiovascular disease; BMI, body-mass index; HbA_1c_, glycated haemoglobin A1c; IHD, ischemic heart disease; LDL-C, low-density lipoprotein-cholesterol; T2D, type 2 diabetes.

**References**

1. Numbero. Cost of Living Index by City 2023 Mid-Year. Accessed 14 December 2024. <https://www.numbeo.com/cost-of-living/rankings.jsp?title=2023-mid>

2. Lui JNM, Lau ESH, Li AQY, et al. Temporal incremental healthcare costs associated with complications in Hong Kong Chinese patients with type 2 diabetes: A prospective study in Joint Asia diabetes evaluation (JADE) Register (2007-2019). *Diabetes Res Clin Pract*. 2025;219:111961. doi:10.1016/j.diabres.2024.111961

3. Appropriate body-mass index for Asian populations and its implications for policy and intervention strategies. *Lancet*. 2004;363(9403):157-63. doi:10.1016/s0140-6736(03)15268-3
